# Discovery of a novel *Betacoronavirus 1*, cpCoV, in goats in China: The new risk of cross-species transmission

**DOI:** 10.1371/journal.ppat.1012974

**Published:** 2025-03-18

**Authors:** Li Mao, Xuhang Cai, Jizong Li, Xia Li, Siyuan Li, Wenliang Li, Honghui Lu, Yichun Dong, Junjun Zhai, Xingang Xu, Bin Li

**Affiliations:** 1 Institute of Veterinary Medicine, Jiangsu Academy of Agricultural Sciences, Nanjing, China; 2 Key Laboratory of Veterinary Biological Engineering and Technology, Ministry of Agriculture and Rural Affairs, Nanjing, China; 3 Jiangsu Key Laboratory for Food Quality and Safety-State Key Laboratory Cultivation Base of Ministry of Science and Technology, Nanjing, China; 4 Jiangsu Co-Innovation Center for the Prevention and Control of Important Animal Infectious Diseases and Zoonoses, Jiangsu Key Laboratory of Zoonoses, Yangzhou University, Yangzhou, China; 5 Guotai (Taizhou) Center of Technology Innovation for Veterinary Biologicals, Taizhou, China; 6 College of Veterinary Medicine, Northwest A&F University, Yangling, China; 7 Animal Husbandry and Veterinary Station of Haimen District, Nantong, China; 8 Animal Husbandry and Veterinary Station of Haian City, Nantong, China; 9 Shaanxi Province Engineering and Technology Research Center of Cashmere Goat, Yulin University, Yulin, China; University of Cambridge, UNITED KINGDOM OF GREAT BRITAIN AND NORTHERN IRELAND

## Abstract

*Betacoronavirus* is a causative agent of respiratory and enteric diseases in humans and animals. Several ruminants are recognized to be intermediate hosts in the transmission of emerging coronaviruses from reservoir hosts to humans. Here, we first report a novel *Betacoronavirus* isolated from goats suffering from diarrhea in China, putatively named caprine coronavirus (cpCoV). Full-genome characterization and nuclear acid comparisons demonstrated that this virus is an evolutionarily distinct *Betacoronavirus* belonging to the subgenus *Embecovirus* and is a *Betacoronavirus* 1 species. Notably, on phylogenetic trees based on complete genomes and RdRp, S, and N genes, the cpCoVs were grouped into a clade distinct from other *Betacoronavirus* strains and were closely related to the HKU23- and HKU23-associated coronaviruses. CpCoV possessed a unique genome organization with a truncated NS4a protein and an elongated NS4b protein that showed no significant matches in the GenBank database. The homology of the S and NS4a-4b genes between cpCoV and *Embecovirus* was less than 95%. Analysis revealed possible recombination events occurred during the evolution of cpCoV and HKU23, and there are striking similarities between the two viruses in evolutionary terms. In addition, cpCoV showed a narrow cell tropism, replicating in human- and bovine-origin cells *in vitro*, and caused diarrhea and enteric pathologic changes in goats and calves *in vivo*. We have provided epidemiological, virological, evolutionary, and experimental evidence that cpCoV is a novel etiological agent for enteric disease in goats. Evidently, a spilling-over event might have occurred between ruminants, including goats, camels, cattle, and wild animals. This study highlights the importance of identifying coronavirus diversity and inter-species transmission in ruminants worldwide, broadens our understanding of the ecology of coronaviruses, and aids in the prevention of animal-to-human transmission and outbreaks.

## Author summary

We report a novel *Betacoronavirus* 1 isolated from goats suffering diarrhea in China, putatively named caprine coronavirus (cpCoV). Compared with previous *Betacoronavirus* 1, cpCoV genome codes a truncated NS4a protein and an elongated NS4b protein, which has no significant matches in the GenBank database, possesses a unique genome organization. In phylogenetical trees, cpCoV grouped into a distinct branch from other *Betacoronavirus* strains and was closely related to the dromedary coronavirus HKU23. Phylogenetic and recombination analyses showed cpCoVs might share a common ancestor with HKU23. Although coronavirus with cpCoV characteristic have not been detected from other ruminants, the S genes of coronaviruses from cattle, dromedary and wild animals shared a close evolutionary relationship with that of cpCoV, and coronavirus spillover events between these ruminants could not be excluded. There is reason to believe that the cross-species transmission might occur between goats and cattle according to the animal models, and the novel virus should be responsible to the severe diarrhea in clinical. The research suggests a high risk of cross-species transmission of coronaviruses among ruminants, highlighting the importance of studying the phylogeny of coronaviruses to understand their evolutionary origins, spillover events and cross-species transmission risk.

## 1. Introduction

Coronaviruses (CoVs), which have been detected in humans and a wide variety of domestic and wild animals, cause respiratory, enteric, and neurological diseases of various severities or asymptomatic infections [1,2]. On the basis of genotypic and serological characterization, CoVs are divided into four distinct groups: *Alphacoronavirus* (α-CoV) and *Betacoronavirus* (β-CoV) mainly infect mammals, while *Gammacoronavirus* (γ-CoV) and *Deltacoronavirus* (δ-CoV) are primarily discovered in birds, with some mammalian spillover [3]. As a result of their unique viral replication mechanism, CoVs have high mutation rates and recombination frequencies [4–8], allowing them to adapt to new host species and ecological niches. Six different CoVs have been identified that affect human beings. Four CoVs (HCoV-OC43, HCoV-NL63, HCoV-229E, and HKU1) induce mild upper respiratory illnesses, with some strains causing severe disease in immunocompetent hosts, infants, young children, and elderly individuals. From 2003, three more highly pathogenic viruses, severe acute respiratory syndrome-related coronavirus (SARS), Middle-East respiratory syndrome-related coronavirus (MERS), and severe acute respiratory syndrome coronavirus 2 (SARS-CoV-2) emerged in humans, causing severe respiratory syndromes. In addition to their pathogenicity to humans, α-CoVs and β-CoVs, including porcine transmissible gastroenteritis virus [9], porcine enteric diarrhea virus [10], bovine coronavirus (BCoV), equine coronavirus, and swine acute diarrhea syndrome coronavirus, also lead to heavy infection burdens in various livestock [11]. Several zoonotic spillover infections and epidemics in humans have been explained by the involvement of intermediate hosts, which create additional complexity when analyzing multi-host species of viruses. SARS-CoV, MERS-CoV, HCoV-NL63, and HCoV-229E are considered to have originated from bats, while HCoV-OC43 and HKU1 are thought to have emerged from rodents [12,13]. Ruminant animals may represent intermediate hosts that allow the transmission of bat-borne or closely related CoVs from wild animals to human beings. It is likely that MERS-CoV spilled over from bats to dromedary camels, and since then has been prevalent in these ruminants [14–17]. In the Arabian Peninsula and Africa, 229E-related CoVs have also been found in dromedary camels, and an analysis of the phylogenetics of complete genomes showed a close evolutionary relationship between dromedary-associated 229E and HCoV-229E [18–20]. The emergence of HCoV-OC43 was postulated to be from bovine CoV, but the zoonotic hosts have not been definitively confirmed. However, the high diversity of OC43-related *Betacoronavirus* 1 strains in livestock species supports the potential role of domestic animals as zoonotic sources in the emergence of HCoV-OC43 [20]. The zoonotic origin of emerging CoVs has yet to be confirmed, and our understanding of CoV diversity and evolution among animals is far from complete. The discovery of novel CoVs in humans and animals is therefore of research interest.

The International Committee on Taxonomy of Viruses has provided a more detailed classification of the genus *Betacoronavirus*, dividing it into five subgroups: *Embecovirus*, *Sarbecovirus*, *Merbecovirus*, *Nobecovirus*, and *Hibecovirus*. This new classification system provides an important framework for understanding β-CoVs. The β-CoV member BCoV belongs to *Betacoronavirus* 1 of the subgenus *Embecovirus*, which also includes HCoV-OC43, HCoV-HKU1, porcine hemagglutinating encephalomyelitis virus, HKU14, HKU24, equine coronavirus (ECoV), mouse hepatitis virus, and canine respiratory coronavirus. BCoV infects calves and adult cattle, which are widespread throughout the world. The virus is transmitted rapidly via the fecal–oral and respiratory routes, including by carrier animals in infected herds [21]. BCoV mainly causes three distinct clinical syndromes, (1) calf diarrhea, (2) winter dysentery with hemorrhagic diarrhea in adults, and (3) respiratory infections in cattle of various ages, including the bovine respiratory disease (BRD) complex or shipping fever of feedlot cattle [22]. BRD is one of the most frequent diseases in the modern beef cattle industry, and is especially detrimental to newly born calves [23,24]. A large number of bovine-like CoVs have been proven to act as potential etiologic pathogens for enteric and/or respiratory diseases in a variety of ruminant species, dogs, and even humans, revealing the possibility of cross-species transmission [3,25,26]. To date, several studies have reported the detection of BCoV in small ruminants such as sheep and goats [26–28], as well as water buffalo [29], dromedary camels [19], alpaca [30], and giraffe [31], and wild animals such as deer [25], water deer [32], and wood bison [33].

Despite the public health and economic significance of BCoVs in cattle and humans, only a limited number of studies have been carried out to evaluate the nature of CoV infections in goats [26]. Although antibodies have been detected in MERS‐CoV-infected sheep and goats, none of the animals effectively shed the virus [16,34,35]. In addition, nasal swabs from domestic mammals, including sheep and goats, in contact with infected dromedaries were demonstrated to have MERS‐CoV nucleic acids [36]. In China, more than 130 million goats were being raised in 2022, which is a large and widely distributed population. Whether goats can be infected by, carry, or transmit CoVs is not known. To provide more information about the potential prevalence and explore the zoonotic origins of emerging CoVs, we performed a systematically and extensively of epidemiology survey for CoVs in goats of China. Here, we report a novel β-CoV, putatively named caprine coronavirus (cpCoV), isolated from goats suffering from diarrhea in China, and provide the first characterization of its occurrence, molecular phylogeny, virus replication, and clinical course, as well as pathohistological changes induced by cpCoV.

## 2. Materials and methods

### 2.1. Ethics statement

The sample collection and animal study were performed in accordance with the institutional animal guidelines and approved by the Ethics Committee of Jiangsu Academy of Agricultural Sciences.

Before sample collection, we contacted local veterinary practitioners and the owners of the animals to obtain their permission. The owners consented to the use and disclosure of questionnaire data for the current study. The collection of all samples and the procedures for the animal experiments were assessed and approved by the ethics committee of the Jiangsu Academy of Agricultural Sciences and performed in strict accordance with the guidelines of Jiangsu Province Animal Regulations (Government Decree No. 45). Each group of experimental animals was kept in one separated room in free-run conditions. All animals were offered water ad libitum and were fed and checked for clinical scores daily during the study period by animal caretakers and study researchers. At the endpoint, all animals were euthanized by humane methods with intramuscular injection of Telazol and intravenous injection of 10% KCl. All procedures were carried out in an approved biosafety laboratory.

### 2.2. Sample collection and handling

**Fig 1:**
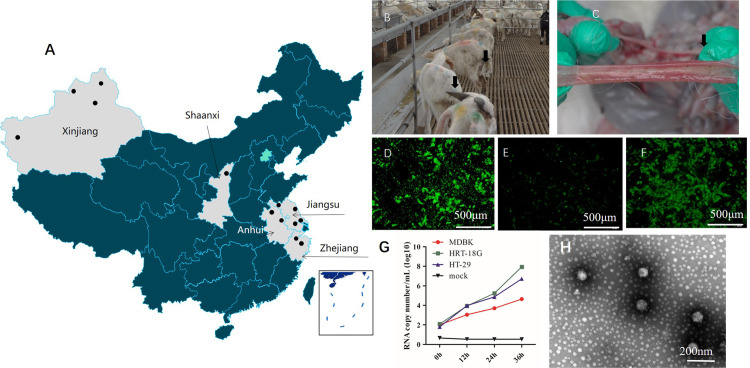
Sample collection site, clinical and pathological observations in infected goats, and the identification of cpCoV. The sample collection sites are marked by dots (The base layer link of the map is from https://vgimap.tianditu.gov.cn/ according to GS(2024)0568)) **(A)**. Diseased goats under 2 months of age showed clinical signs, including depression symptoms, varying degrees of diarrhea, weight loss **(B)**, and intestinal tract bleeding **(C)**. IFA detection of cpCoV-infected HRT-18G **(D)**, MDBK, **(E)** and HT-29 **(F)** cells by anti-Spike protein monoclonal antibody. Replication of cpCoV/AHFY2302G in HRT-18G, HT-29 and MDBK cells. Cells were infected with isolate AHFY2302G and harvested at 0-36 hpi, and replication was measured by qRT-PCR **(G)**. Transmission electron micrograph of cpCoVs using negative staining with 2% sodium phosphotungstate. Coronaviruses were observed as typical corona-shaped particles of 70–90 nm in diameter **(H)**.

From March 2022 to December 2023, 1,172 rectal swabs were collected from diseased goats in the Jiangsu, Anhui, Zhejiang, Shaanxi, and Xinjiang provinces/autonomous regions of China (Fig 1A). The goats showed symptoms of gastrointestinal infections, with varying degrees of diarrhea severity (Fig 1B and 1C). All swab samples were collected and recorded by professional veterinarians and stored at −80 °C.

### 2.3. CoV detection by PCR

Total RNA was extracted from swabs and tissues using a EasyPure Simple Viral DNA/RNA Kit (Transgen, Beijing, China), following the manufacturer’s instructions, and used for reverse transcription (RT)-PCR. The spike (S), nucleocapsid (N), and NS4a-4b genes were amplified by specific primers (S1 Table). Due to the NS4a-4b gene sequences being significantly different between caprine and bovine coronaviruses, the two viruses could be clearly distinguished by sequencing. In brief, RT was conducted using a SuperScript III kit (Takara, Dalian, China). The PCR mixture (25 μL) was performed as follows: 1 μL of cDNA, 1 μL each of forward and reverse primers (10 μM), 12.5 μL of Q5 High-Fidelity 2× Master Mix (Takara), and 9.5 μL of nuclease-free water. Amplification involved 98 °C for 30 s, 35 cycles at 98 °C for 10 s, 56 °C for 30 s, and 72 °C for 1 min, with a final extension step at 72 °C for 5 min in an automated thermal cycler (BIO-RAD, CA, USA). The PCR products were recycled, purified, and ligated to pMD-18-T (Takara), and positive plasmids were sent for sequencing (Tsingke Biotechnology Co., Ltd., Nanjing, China).

### 2.4. Real-time PCR quantitation

Real-time quantitation PCR (qRT-PCR) was performed on samples and cell cultures to detect cpCoV. Total nucleic acids from samples and cells were sent for analysis via TaqMan qRT-PCR assay. Briefly, the qRT-PCR was performed with an ABI Step One system (Applied Biosystems, Foster City, CA, USA) using the HiScript II One Step qRT-PCR Probe Kit (Vazyme Biotech Co., Ltd., Nanjing, China), which included 100 nM of each primer BCoV-qF/BCoV-qR and BCoV-q-probe (detailed in S1 Table), 4 μL of RNA template, and deionized distilled water. The TaqMan qRT-PCR conditions were as follows: reverse transcription at 50 °C for 5 min, initial denaturation at 95 °C for 30 s, 40 cycles at 95 °C for 10 s, and final extension at 60 °C for 34 s. The sensitivity of this method was up to 10 copies per mL.

### 2.5. Virus isolation

The following 10 cell lines, HRT-18G (human rectum epithelial), HT-9 (human T lymphoid cell), MDBK (bovine kidney), Marc145 (monkey embryonic kidney), Vero (African monkey kidney), 293T (human kidney), MA104 (monkey embryonic kidney), IPEC-J2 (pig intestine), FK18 (feline kidney), and BHK-21 (baby hamster kidney), were cultured for virus isolation in this study. All cell lines were obtained from ATCC and stored in our laboratory, and tested as being free of CoV, bovine viral diarrhea virus (BVDV), and mycoplasma. Extracts from PCR-positive rectal swabs were centrifuged at 10,000 rpm for 30 min and filtered with 0.22 μm filters before culture with cells. After a 1 h incubation at 37 °C, the inoculum was removed and replaced with serum-free Dulbecco modified Eagle medium (DMEM, Gibco, Shanghai, China) with trypsin supplementation (1 g/mL) (Sigma, St. Louis, MO, USA). All infected cell lines were incubated at 37 °C for 4 days and observed for cytopathic effects (CPEs). After three blind passages, the supernatant and cell pellet were examined for the presence of the virus by qRT-PCR targeting the N gene and RT-PCR targeting the S and NS4a-4b genes.

Angiotensin-converting enzyme 2 (ACE2), aminopeptidase N (APN), and dipeptidyl peptidase 4 (DPP4) are well-known cellular receptors for coronaviruses [37–39]. Cell lines stably expressing human (h) APN and ACE2 were employed to test the specific receptors used by the novel coronaviruses. The 293T cells expressing hACE2 (293T-hACE2) (Protein Data Bank 2AJF) were gifted generously by Prof. Zhen Liu of Nanjing University, and hACE2 expression was analyzed by indirect immunofluorescence assay (IFA) with ACE2 antibodies (ABclonal, Wuhan, China). The BHK-21-hAPN cell line was constructed in our lab. Briefly, APN wild-type (GenBank: M22324.1) was cloned into pCDNA 4.0 plasmid with a C-terminal Flag tag, then used to transfect BHK-21 cells. After treatment with 300 μg/mL Zeocin (Invitrogen, Shanghai, China) for 1 week, single-colony cells were collected and harvested for analysis of protein expression by IFA as described previously [40]. The isolated cpCoVs were inoculated into cultures of 293T cells expressing 293T-hACE2 and BHK-21-hAPN and cultured for 24 h. The cells were fixed and subjected to indirect immunofluorescence assay (IFA) with a monoclonal antibody against BCoV S1 protein (prepared in our lab).

### 2.6. Neutralization assays

Briefly, goat or calve serum was serially diluted 1:2 and then mixed with 100 50% tissue culture infective doses (TCID_50_) of cpCoV AHFY2302G. After incubation for 1 h at 37 °C, the mixture was inoculated in duplicate onto 96-well plates of HRT-18G cell cultures. CPEs were observed and recorded after 4 days of incubation. Serum samples were tested for the presence of cpCoV-reactive antibodies with a cut-off of 1/16.

### 2.7. Electron microscopy

Negative-contrast electron microscopy was performed as described previously [41]. After being infected with cpCoV AHFY2302G, cell culture extracts were centrifuged at 19,000 × *g* at 4°C for 30 min, followed by concentration at 35,000 × *g* for 30 min and 120,000 × *g* for 2 h in a Beckman Optima L-100XP ultracentrifuge with a 70Ti rotor. The pellet was resuspended in phosphate-buffered saline (PBS) and stained with 2% phosphotungstic acid. The stained grids were air-dried, and virus particles were visualized with a transmission electron microscope (TEM) (Hitachi HT7800, Japan).

### 2.8. IFA

IFA was used to detect cpCoV infection and ACE2 and APN expression. Briefly, after washing with PBS, the cells were fixed with 4% paraformaldehyde and incubated with 1% bovine serum albumin (BSA) (Shanghai Macklin Biochemical Technology Co., Ltd., Shanghai, China) for 1 h respectively. The permeabilized cells were then incubated with antibodies (CoV-S1-4 monoclonal antibody, prepared and stored in our laboratory, was reacted with BCoV and cpCoV; anti-human APN and anti-human ACE2 monoclonal antibodies, Abclonal). Then, the cells were washed with PBS and stained with fluorescein isothiocyanate (FITC)-labelled mouse anti-rabbit antibody (Boster Biological Technology Co. Ltd., Wuhan, China) and 4′,6-diamidino-2-phenylindole, dihydrochloride (DAPI, Beyotime, Shanghai, China). Observation and image acquisition were conducted under inverted fluorescence microscope (Olympus, Tokyo, Japan).

### 2.9. Complete genome sequencing of novel CoV from goats

To characterize the novel CoV, CoV-positive cell cultures were pretreated for next-generation sequencing (NGS) (Illumina HiSeq2500). The sequencing procedure was conducted by Shanghai Tanpu Bio company (Shanghai, China). The full-length genomes of caprine-origin CoV were assembled by Illumina sequencing technology. Briefly, DNase treatment and cleanup were conducted before library preparation using Nextera XT reagents and sequencing on the NovaSeq 6000 (Illumina). After read quality trimming and an additional trimming filter for unreliable sequences, host read subtraction was carried out with the BWASW program against ribosomal RNA (16, 18, 23, 28, 5S, and internal transcribed spacer rRNA), bacterial genome, and host organism genome sequences. SPAdes and MEGAHIT software were used to *de novo* assemble the reads, and final scaffolds were subjected to BWASW read mapping and a mega blast homology search against the NCBI NT database.

### 2.10. Phylogenetic analysis

To clarify the evolutionary relationships between the novel CoV and previously identified CoVs, we conducted nucleotide sequence alignments of complete CoV sequences using ClustalX software (http://www.clustal.org). Multiple sequence alignments of the viral genome and S, RNA-dependent RNA polymerase (RdRp), and N genes were carried out using ClustalW in the MegAlign program (DNAStar software), together with reference sequences from representative CoV strains. Molecular phylogenetic trees were constructed using the neighbor-joining method with MEGA 7.0 software. A total of 1,000 bootstrap replicates were used to derive the trees based on the nucleotide sequences of the genome segments. To detect possible recombination events, bootscan analysis with Simplot program version 3.5.1 was used to identify recombination points within the novel cpCoV genome sequences, with a window size of 1,000 bp, moving in 200-nucleotide steps with 1,000 bootstrap values and a confidence threshold of 90%.

### 2.11. Animal experiment design

Samples from six 2-month-old healthy goats and 12 newborn calves (Xuyi Weigang Animal Husbandry Co., Ltd., a commercial breeding farm with a high veterinary hygiene standard) were analyzed for cpCoV, rotavirus (RV), bovine enterovirus, BVDV, and astrovirus by qRT-PCR and a virus neutralization assay for the detection of antibodies against cpCoV. The mothers of the calves were tested for cpCoV, BVDV, RV, and antibodies against cpCoV. All animals tested negative for the viral antigens and cpCoV antibodies before the experiment. Newborn Holstein calves born within 48 h received in a single shipment were used in this study. At the start of the study, all animals were of similar weight, randomly assigned to the challenge or control groups, and housed in the same building; negative control animals were kept in a separate room.

Six goats were randomly divided into two groups. For the challenge group (CC-goat), three goats were infected intranasally with 5 × 10⁵ TCID_50_ cpCoV/AHFY2302G. For the control group (NC-goat), three animals were infected intranasally with the same volume of DMEM medium. At 12 days post-infection (dpi), the goats in the two groups were euthanized by humane injection. The calves were divided into two groups, with 6 animals in each group. Six calves (CC-calf) were infected with 2 × 10^6^ TCID_50_ cpCoV/AHFY2302G intranasally, and the other six were used as controls (NC-calf) and infected intranasally with the same volume of DMEM medium. At 7 dpi and 14 dpi, three inoculated calves and three control animals were euthanized and grouped as NC-7, CC-7, NC-14, and CC-14. The rectal temperature (normal temperature for goats is 38.5 °C–39.7 °C and calves is 38.5 °C–39.5 °C) and clinical symptoms, including cough, nasal discharge, diarrhea, food intake, and mental status, were monitored throughout the animal experiment. Viral RNA shedding was detected in sera (collected by ear vein puncture) and nasal, rectal, and oral swabs every day under conditions of humanitarian care and adequate reassurance of the animal from infection to endpoint. For virus load detection and histopathology, all animals were subjected to autopsy, and the tissues of the heart, liver, spleen, lung, renal, trachea, lymph nodes, duodenum, jejunum, ileum, cecum, colon, and rectum were collected. Diarrhea symptoms were recorded daily in each group according to the following scoring system: no diarrhea = 0, sticky feces = 1, loose feces = 2, and watery feces = 3. At autopsy, tissues were fixed in 10% formalin, embedded in paraffin, then cut into 3-μm sections and stained with hematoxylin and eosin.

## 3. Results

### 3.1. Animal surveillance and identification of a novel *Betacoronavirus* 1 from goats

Since 2022, several outbreaks of disease have occurred on goat farms in China. Clinical signs, including depression symptoms and gastrointestinal infections with varying degrees of diarrhea, weight loss, and death, were observed in goats under 2 months of age (Fig 1A–C). In total, 1,172 rectal swabs were collected from diseased goats in Jiangsu, Anhui, Zhejiang, Shaanxi, and Xinjiang provinces/autonomous regions of China during 2022–2023. Of these, 196 (16.7%) samples tested CoV-positive by qPCR, with the positive detection rate reaching 80% (24/30) in a farm with a severe outbreak of diarrhea. Samples from coronavirus-infected goats were sent for NGS, which showed the strain diverged from currently characterized CoVs, especially in the sequences NS4a-NS4b. We designed an RT-PCR targeting NS4a-NS4b to differentiate cpCoV from BCoV for a subsequent epidemiological sequencing investigation. The cpCoV percentage was higher in regions of eastern China, namely Anhui (26.7%; 62/232), Jiangsu (21.7%; 77/355), and Zhejiang (11.6%; 15/129), and lower in regions of northwest China, namely Xinjiang (9.2%; 25/273) and Shaanxi (9.3%; 17/183) (Table 1), which are the main areas for goat production. To our knowledge, this study was the first to provide epidemiologic information on cpCoV infections among goats in different regions of China. Our findings indicate that the novel CoV is a pathogen associated with the occurrence of enteric disease in goats.

**Table 1:** Detection and isolation of caprine coronavirus in goat samples from different provinces/autonomous regions of China.

Province/autonomous region	City	Sampling site	Samples	cpCoV-positive	Percentage (%)	Isolates
Jiangsu			355	77	21.7	HMsz2207, RG23F3, YC2304G, Haian/4, Haian/5-4, RG23/H2, RG23/G1
	Yancheng	4	122	23	18.9	
	Changzhou	2	48	9	18.8	
	Nantong	7	108	34	31.5	
	Xuzhou	3	77	11	14.3	
Anhui			232	62	26.7	AHFY2302G
	Fuyang	5	140	46	32.9	
	Chuzhou	6	182	16	8.8	
Zhejiang			129	15	11.6	ZJ2303G, ZJ23/G1, ZJ23/GAN
	Jinhua	2	29	8	27.6	
	Huzhou	3	78	7	9.0	
	Hangzhou	1	22	0	0	
Shaanxi			183	17	9.3	/
	Yulin	7	183	17	9.3	
Xinjiang			273	25	9.2	XJCJ2301G
	Changji	6	85	16	18.8	
	Aletai	4	60	7	11.7	
	Tacheng	6	72	0	0	
	Kashi	5	56	2	3.6	
Total			1172	196	16.7	

### 3.2. The novel cpCoV had narrow cell tropism, and human APN and ACE2 were not receptors for the virus

In total, 12 different cpCoVs were isolated from Jiangsu, Anhui, Zhejiang, and Xinjiang provinces/autonomous regions (Table 1) using HRT-18G cell lines in the presence of trypsin. In HRT-18G cells, a CPE in the form of rounded, fused, and granulated giant cells that rapidly detached from the monolayer was clearly observed from 48 h after inoculation. The viral S protein was detected by an indirect IFA using the specific monoclonal antibody CoV-S1-4 (Fig 1D), while mock infected cells showed negative results. For six of these strains (HMsz2207, RG23F3, YC2304G, AHFY2302G, XJCJ2301G, and ZJ2303G), complete genomic sequences were obtained (OR077306–OR077311). For the remaining six strains (Haian/4, Haian/5-4, RG23/H2, RG23/G1, ZJ23/G1, ZJ23/GAN), S-NS5a region sequences were obtained, which were submitted to the GenBank database (PP174288–PP174293). Ten cell lines were inoculated with cpCoV and blind cultured for three passages, and virus was detected by IFA and qRT-PCR in the cultures of HRT-18G, HT-29, and MDBK cells (Figs 1D–1G and S1 and S3 Table). In these three cell line cultures, the log copy numbers of the target CoV RNA increased throughout the infection (Fig 1G and S3 Table), and significant specific fluorescence was observed in IFA assay. The S-NS5a region was amplified by RT-PCR and sequenced, which confirmed the cpCoV isolation. CpCoV replicated efficiently *in vitro* in the HRT-18G and HT-29 cell lines (Fig 1D, 1F, and 1G), and the CT values in the qRT-PCR were <16. Only minimal CPE, with a few cells showing rounding and weak fluorescence in the IFA assay, was observed for the cpCoV-infected MDBK cells (Fig 1E), and the CT values obtained by qRT-PCR were between 24.2 and 32.6. According to the IFA and qRT-PCR results, Marc145, Vero, 293T, MA104, IPEC-J2, FK18, and BHK-21 cells did not support the growth of cpCoV. To further verify that the detected virus was indeed a coronavirus, the ultracentrifuged cell culture extracts from AHFY2302G-infected HRT-18G cells were sent for TEM observation. Classical CoV particles of ~70 nm to 90 nm in diameter, with typical corona-shaped surface projections, were present (Fig 1H). The particle sizes and morphology were compatible with the ranges described for CoVs [21].

Known coronavirus host cell receptors include ACE2 for SARS-related CoV and APN for certain alphacoronaviruses [38,42] such as human CoV-229. To investigate the receptor usage of cpCoV, we studied the live cpCoV infection of 293T-hACE2 and BHK-21-hAPN cells that expressed the two receptor molecules (S2A–S2C and S3A–S3C Figs). The positive control showed SARS-CoV-2 pseudoviruses could bind to ACE2 receptors (S2D Fig), but the IFA and qRT-PCR results provided no evidence of enhanced infection or entry into hACE2- or hAPN-expressing cells by cpCoVs (S2 and S3 Figs), suggesting that none of these surface molecules function as entry receptors for the novel virus.

### 3.3. Genome organization and coding potential of the novel *Betacoronavirus*

**Fig 2:**
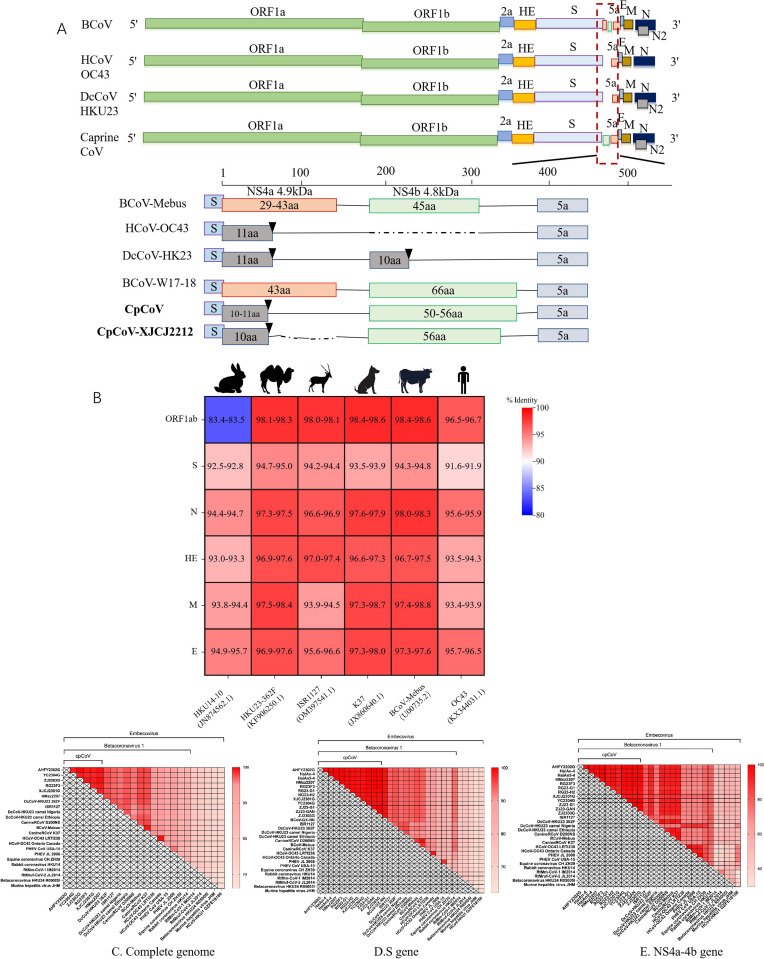
(A) Genomic organization of cpCoV and other β-CoV strains in the region between the Spike (S) gene and the NS5a gene. Distinct open reading frame (ORF) patterns were detected in β-CoV 1 in this region. Black triangles indicate stop codons. Horizontal dotted lines indicate a deletion. **(B)** Nuclear acid comparison of *Embecovirus* ORF1ab and structural proteins. CpCoVs were used as reference. Numbers and colors in the heat map represent the percentage identities between selected strains and cpCoVs. Pairwise identity scores were generated using MegAlign software, and the heat map was generated in Prism software. Heatmap of homology analysis based on the complete genome **(C)**, and the S **(D)** and NS4a-4b **(E)** genes of cpCoV and reference strains.

The cpCoV genomes were determined to be approximately 31,005 nucleotides in length (AHFY2302G as a reference strain), with a genomic organization highly similar to that of BCoV: 5′-UTR (nucleotide (nt) 1–198), ORF1ab (nt 199–21482), ORF2a (nt 21492–22328), hemagglutinin esterase (nt 22340–23614), S (nt 23629–27720), NS5a (nt 28083–28412), envelope (E) (nt 28399–28653), membrane (M) (nt 28668–29360), N (nt 29370–30716), and 3′-UTR (nt 30717–31005) (Fig 2A). The cpCoV complete genome sequences were compared with *Embecovirus* reference sequences. The nucleotide identity of the complete genome sequences of the six cpCoV isolates was 98.9%–99.9%, and with the reference sequence, it was 72.1%–98.6% (Fig 2C). The nuclear acid sequence of coronavirus ORF1ab and its major structural protein genes were compared with *Betacoronavirus 1* from animals (Fig 2B). For ORF1ab, N, and M genes, cpCoVs are more similar to BCoV Mebus than to the other *Embecoviruses*, with pairwise identities of 98.4%–98.6%, 98.0%–98.3%, and 97.4%–98.8%, respectively. For major structural protein genes S and hemagglutinin-esterase, cpCoVs were most similar to DcCoV HKU23, with 94.7%–95.0% and 96.9%–97.6% similarity, respectively. The coding potential and characteristics of putative nonstructural proteins (nsp) of ORF1 of cpCoVs are shown in S2 Table. The ORF1 polyprotein possessed >99% amino acid (aa) sequence identity to the corresponding sequences of the BCoV and HKU23 polyproteins. The lengths, predicted cleavage sites, and seven conserved replicase domains for CoV species demarcation, i.e., nsp3 (ADP-ribose 1“-phosphatase, ADRP), nsp5 (3C-like protease, 3CL^pro^), nsp12 (RdRp), nsp13 (Hel), nsp14 (exonuclease, ExoN), nsp15 (nidoviral uridylate-speciﬁc endoribonuclease, NendoU), and nsp16 (2′-*O*-ribose methyltransferase, O-MT), were conserved between cpCoV and the corresponding nsp in strains of *Betacoronavirus 1* (BCoV, HKU23, and canine respiratory CoV).

The major differences in sequence identity among the isolates were within the S and NS4a-4b genes. The homology of the S and NS4a-4b gene sequences between the 12 cpCoV strains was 98.8%–100% and 91.6%–100%, respectively, while the homology between cpCoV and *Embecovirus* was 66.7%–95% and 54.7%–95.3% (Fig 2D–2E), respectively. CpCoV showed a unique genomic organization in a 400-nt region between the S and NS5a genes. In this region, two small open reading frames (ORFs) were identified in BCoV, NS4a and NS4b, encoding 4.9- kDa and 4.8-kDa nsps, respectively, previously suggested to be vestiges of an 11-kDa protein encoded by the mouse hepatitis virus. Sequence alignment of this region with BCoV Mebus revealed two deletions in cpCoV. The cpCoV NS4a proteins contained only 10–11 aa, compared with the 43 aa protein of BCoV Mebus, which is due to a 5-nt deletion leading to a premature stop codon. The NS4b protein of cpCoV was 50–56 aa in length because of a 10-nt deletion not seen in other BCoVs (S4 Fig). A pairwise comparison of the NS4a-4b region among CoVs, including BCoV-Mebus, HCoV OC43, dromedary camel coronavirus HKU23 (DcCoV-HKU23) strain 362F, ISR1127 from *Oryx leucoryx* [43], and water deer coronavirus W17-18 [32], revealed the distinct genomic organization of the NS4a and NS4b sequences of cpCoV compared with those of other *Embecovirus* members. A BLAST analysis of the cpCoV NS4a and NS4b proteins returned no significant matches in the database. Strain XJCJ2304G even harbored a 54-nt deletion between the NS4a and NS4b genes, similar to rabbit coronavirus HKU14 (S4 Fig) [44]. The stepwise deletion patterns of NS4a and 4b observed in BCoV, DcCoV-HKU23, cpCoV, cpCoV/XJCJ2212, RbCoV-HKU14, and HCoV-OC43 may indicate the evolutionary relationships between the viruses.

### 3.4. Phylogenetic analyses grouped the cpCoVs into a separate clade that shared close relationship with dromedary coronavirus HKU23

**Fig 3:**
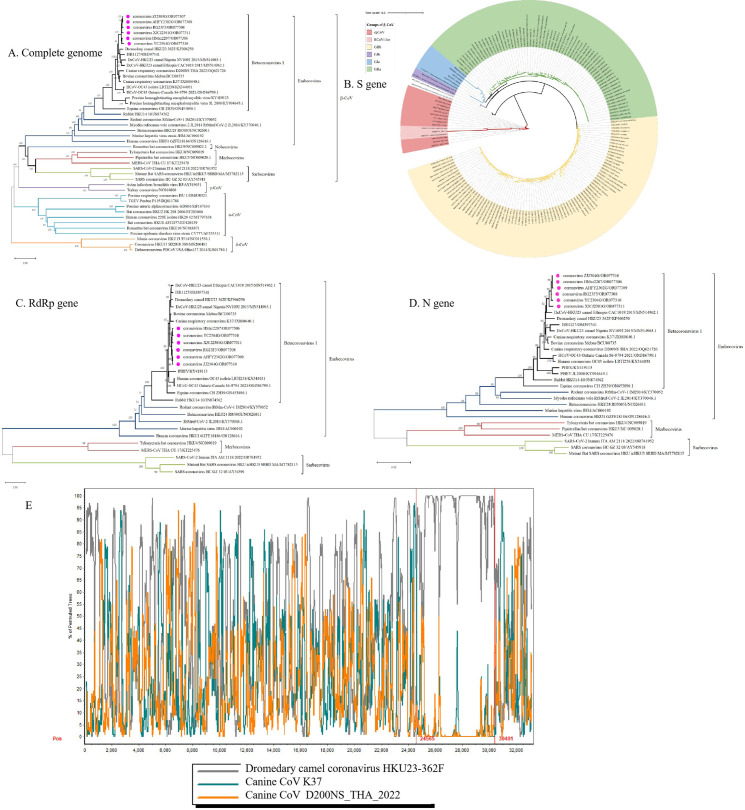
Phylogenetic tree based on the complete genome **(A)**, S gene **(B)**, RNA-dependent RNA polymerase (RdRp) **(C)**, and N gene (1,347 nt) **(D)** of cpCoV and representative CoVs. The alignment of each gene was manually trimmed to 31,005 nucleotides (nt) for the whole genome, 4,092 nt for the S gene, 2,783 nt for RdRp, and 1,347 nt for the N gene. Analyses were conducted using MEGA software version 6.0 (http://www.megasoftware.net) with the neighbor-joining algorithm. Bootstrap values (indicated on each branch) were calculated with 1,000 replicates. Circles indicate the cpCoV strains. Scale bar = nucleotide substitutions per site. CoV, coronavirus; PDCoV, porcine deltacoronavirus; PEDV, porcine epidemic diarrhea virus; PHEV, porcine hemagglutinating encephalomyelitis virus; PRCV, porcine respiratory coronavirus; SARS, severe acute respiratory syndrome; TGEV, transmissible gastroenteritis virus. **(E)** Recombination analysis of the genomes of cpCoV/AHFY2302G, DcCoV-HKU23, canine CoV D200NS_THA_2022, and K39. Bootscan analysis was performed with Simplot, version 3.5.1.

Phylogenetic analyses of the complete genome (Fig 3A) and S (Fig 3B), RdRp (Fig 3C), and N (Fig 3D) gene sequences were conducted using the neighbor-joining method. In the complete genome, RdRp, and N trees, cpCoVs formed a distinct cluster within the β-CoV subgroup *Embecovirus* and were more closely related to HKU23 from dromedary camels and ISR1127 from *Oryx leucoryx* than BCoV and other bovine-like CoVs, despite the geographic distance between cpCoV isolations [45]. On phylogenetic analysis of the S gene, the cpCoVs appeared to be genetically distinct from almost all BCoVs and BCoV-like strains. They were grouped into a clade with strain BCoV-GX-NN190313, DcCoV-HKU23/362F, and *Oryx leucoryx* CoV that was separate from that of previously identified *Embecovirus,* and shared a closer relationship with HKU23 viruses than other β-CoVs (Fig 3B). Homology analysis also showed that the S gene of cpCoVs shared high homology (94.7%–95.0%) with that of HKU23/362F (GenBank accession: KF906250) (Fig 2B). To determine the genus and species that caprine coronavirus belongs to, we followed ICTV criteria [46], and our results suggest that cpCoV is a novel member of the *Betacoronavirus* genus, *Embecovirus* subgenus, and belongs to the *Betacoronavirus* 1 species.

### 3.5. Recombination during the evolution of the cpCoV genome

To determine whether recombination had occurred during the evolution of cpCoV, we aligned the genomic sequences of cpCoV/AHFY2302G, camel CoV HKU23-362F, canine CoV-D200NS_THA_2022, and K39. After examining the results, a recombination event was discovered in the S and N gene regions (Fig 3E). Bootscan analysis showed possible recombination sites in the 24565–30401 regions of the cpCoV, which strong evidence of cross-species transmission.

### 3.6. CpCoV infection linked to diarrhea disease in goats

**Fig 4:**
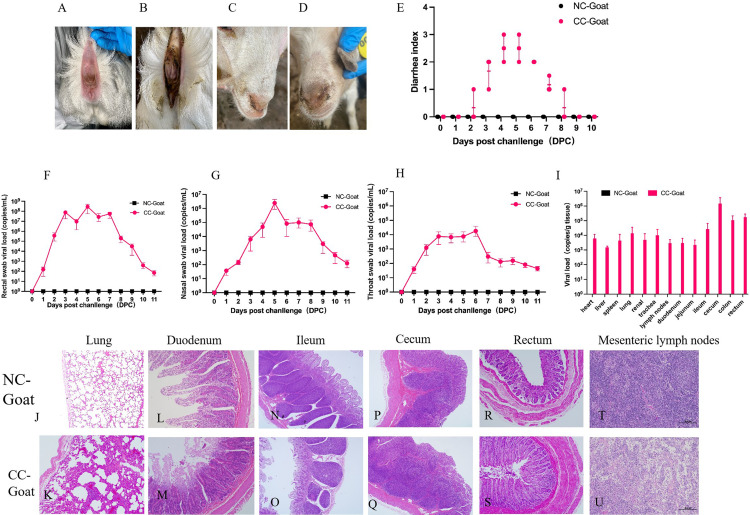
Pathogenicity experiments in cpCoV-infected goats. Goats were intranasally inoculated with 5 × 10^5^ TCID_50_ virus. Diarrhea **(B)** and nasal discharge **(D)** were observed in goats in the challenge groups (CC-Goat), whereas the control group (NC-Goat) goats were normal (**A** and **C**). **(E)** Diarrhea index was evaluated in the CC-Goat and NC-Goat groups; the goats in the CC-Goat group showed apparent diarrhea. CpCoV viral RNA shedding was detected in rectal **(F)**, nasal **(G)**, and throat **(H)** swabs. The viral load was detected in almost all organs of goats (**I)**. Histopathological examination of cpCoV-infected goats. The alveolar wall of the CC-Goat group was significantly thickened, epithelial cells were shed, and the alveolar cavity was filled with lymphocytes (**J** and **K**). The duodenum (**L** and **M**), ileum (**N** and **O**), cecum (**P** and **Q**), and rectum (**R** and **S**) had different degrees of villus atrophy and severe mucosa abscission. Lymphocytes in mesenteric lymph nodes were markedly reduced and sparse (**T** and **U**).

In the cpCoV challenge group, the goats developed significant symptoms of diarrhea (Fig 4A and B) and nasal discharge (Fig 4C and 4D) from 2 dpi to 8 dpi. Their feces appeared soft and shapeless, there was occasional watery diarrhea without blood, the diarrhea scores increased progressively (Fig 4E and S4 Table), but rectal temperatures remained normal (38.7 °C–39.3 °C). No clinical signs, fever, diarrhea, or mortality were observed in the control group. In the rectal, nasal, and oral swabs, cpCoV RNA shedding was observed in all three infected goats from 1 dpi to 11 dpi (Fig 4F–4H and S5–S7 Tables). Fecal and nasal shedding was observed in all three goats between 1 dpi and 10 dpi, peaking between 3 dpi and 7 dpi, with quantification ranging from 1.45 × 10^6^ to 5.05 × 10^8^ and 9.24 × 10^4^ to 4.47 × 10^6^ genome copies per mL, respectively, then decreasing significantly after 11 dpi. Rectal swabs had the highest viral load. The viral load of throat swabs was lower than that of nasal and rectal swabs, and at 2–7 dpi was quantified as < 3.82 × 10^4^ genome copies per mL.

The goats in the challenge and control groups were euthanized on day 12, and cpCoV was detected in almost all of the collected tissues by RT-qPCR, including the heart, liver, spleen, lung, renal, trachea, lymph nodes, duodenum, jejunum, ileum, cecum, colon, and rectum, with higher viral loads in the cecum, colon, and rectum (2.26 × 10^5^ to 3.92 × 10^6^, 1.34 × 10^4^ to 2.20 × 10^5^, and 7.0 × 10^4^ to 2.83 × 10^5^ genome copies per g, respectively) than other tissues (Fig 4I and S7 Table). Interestingly, viral RNA was not detected in the sera of the challenge group, and no cpCoV shedding was detected in any tissue samples from the control group. Necropsy of goats showed that the lesions in the challenge group were mainly concentrated in the intestine. Compared with the mock group, the challenge group’s small intestine walls had obviously thinned, and bleeding was observed at the intestinal mucosa. Histopathological examination showed that the alveolar wall of the challenge group was significantly thickened, epithelial cells were being shed, and the alveolar cavity was filled with lymphocytes (Fig 4J and 4K). The duodenum (Fig 4L and 4M), ileum (Fig 4N and 4O), cecum (Fig 4P and 4Q), and rectum (Fig 4R and 4S) showed different degrees of villus atrophy and severe mucosa abscission. Lymphocytes in the mesenteric lymph nodes were markedly reduced and sparse (Fig 4T and 4U).

The fecal samples (3–7 dpi) and tissue samples (cecum, colon, and rectum) from goats in the challenge group and negative control group were inoculated into HRT-18G cells. After three blind passages, the challenge group samples induced a CPE, and the cell cultures were identified as cpCoV-positive by RT-qPCR, RT-PCR, and IFA methods. NS4a-5a sequencing confirmed the isolation of the virus. In contrast, the negative control samples showed negative results. These findings indicated that the animal challenge experiments with cpCoV adhered to Koch’s postulates, and cpCoV is an important pathogen associated with diarrhea disease in goats.

### 3.7. CpCoV infection caused severe diarrhea in calves

**Fig 5:**
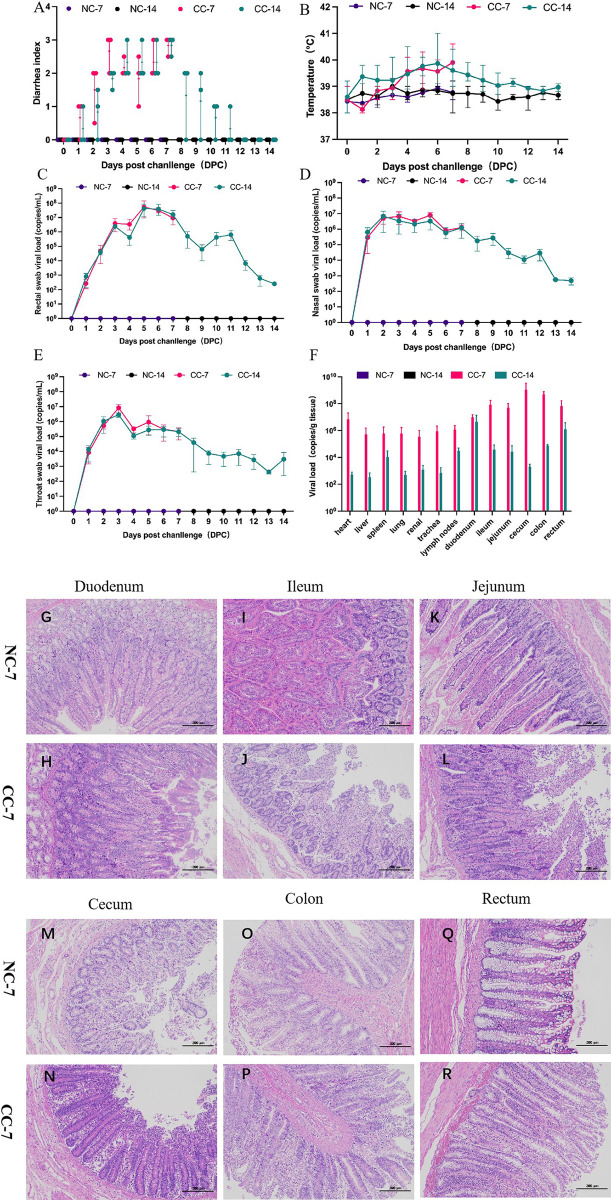
Pathogenicity experiments in cpCoV-infected calves. The animals were intranasally inoculated with 2 × 10^6^ TCID_50_ virus. The diarrhea index was evaluated in the four groups. Calves in the CC-7 and CC-14 groups showed apparent diarrhea **(A)** and an elevated temperature **(B)** compared with the NC groups. CpCoV viral RNA shedding was detected in rectal **(C)**, nasal **(D)**, and throat **(E)** swabs. Viral load was detected in almost all organs of calves in CC-7 and CC-14 groups **(F)**. Histopathological examination of cpCoV-infected calves. Pathological changes in calves in the CC-7 group were mainly in the intestine, with severe disintegration and shedding of the intestinal villi in the duodenum (**G** and **H**), ileum (**I** and **J**), jejunum (**K** and **L**), cecum (**M** and **N**), colon (**O** and **P**), and rectum (**Q** and **R**).

The 12 calves used for the cpCoV infection experiments were divided into four groups: NC-7, NC-14, CC-7, and CC-14. At 1 dpi, challenged calves (3/6) showed mild symptoms of diarrhea and sticky feces. At 3 dpi, all calves developed various degrees of diarrhea (100% incidence rate), accompanied by poor appetite and depression. Diarrhea and even bloody stools were observed in all challenged calves at 3–8 dpi (Fig 5A and S9 Table), and three of the calves with severe diarrhea were euthanized at 7 dpi. From 8 dpi to 11 dpi, the diarrhea in the remaining three challenged calves gradually subsided, and two of the calves recovered normal defecation. At 12–14 dpi, the defecation of all animals returned to normal, and their psychiatric status and appetite improved significantly. The average body temperature of the challenge group (38.5 °C–40.5 °C) was 1 °C higher than that of the control group (38.0 °C–39.3 °C) (Fig 5B and S10 Table). With the aggravation of diarrhea symptoms, the rectal temperature of the cpCoV-infected calves increased significantly, then normalized as diarrhea symptoms relieved, while that of calves in the control group remained normal, and they showed no clinical signs or increases in body temperature.

CpCoV-infected calves showed evidence of viral shedding in their nasal, throat, and rectal swabs. While the viral load of the nasal and throat swabs had increased significantly at 1 dpi, that of rectal swabs increased more slowly (Fig 5C–5E and S11–S13 Tables). Viral RNA shedding could be detected in all swabs from 1 dpi to 14 dpi. The viral load was significantly higher in rectal swabs than nasal and throat swabs, reaching 1.43 × 10^8^ copies/mL at 5 dpi. Nasal and throat viral shedding increased slowly from 1 dpi to 3 dpi, followed by a gradual decline from 4 dpi to 10 dpi. During the infection, viremia was not observed in the challenge group, similar to the observations of cpCoV-infected goats.

The NC-7 and CC-7, NC-14, and CC-14 groups of calves that were euthanized at 7 dpi and 14 dpi tested positive for the virus when RT-qPCR was performed on various tissue samples (heart, liver, spleen, lung, renal, trachea, lymph nodes, duodenum, jejunum, ileum, cecum, colon, rectum), with higher viral loads in the cecum, colon, and rectum in CC-7 group (3.89 × 10^6^ to 3.27 × 10^9^, 8.33 × 10^6^ to 7.81 × 10^8^, 1.36 × 10^5^ to 1.64 × 10^8^ genome copies per g, respectively) than that of NC-7 (Fig 5F and S14 Table). The degree of viral loading in the CC-14 group of calves euthanized at 14 dpi was significantly lower than that of the CC-7 group. No cpCoV RNA was detected in any tissue samples from calves in the negative control (NC) group. The results indicated that cpCoV mainly affected the intestines of the calves, which is consistent with the observed clinical manifestations. According to the detection results, the tissue viral load in the CC-7 group calves was approximately 10^1.5^ to 10^5^ times higher than that in the CC-14 group, and there was obviously difference in the cecal viral load between the two groups of challenged calves. The viral load also corresponded to the severity of the presenting symptoms.

All six calves in the challenge group showed an enlargement of the mesenteric lymph nodes, mesenteric congestion, and obvious thinning of the whole small intestine. The cecum was inflated, and multiple hemorrhage sites in the small and large intestine walls were observed at necropsy. No abnormalities were observed in calves in the control group. Pathological changes in tissue sections from calves in the cpCoV challenge group were mainly concentrated in the intestine, with severe disintegration and shedding of the intestinal villi in the duodenum, jejunum, ileum, cecum, colon, and rectum (Fig 5G–5R). The results showed that cpCoV could infect calves by cross-species transmission and cause severe diarrhea and respiratory symptoms.

## 4. Discussion and conclusion

We discovered a novel *Betacoronavirus* 1, cpCoV, infecting goats in China. To our knowledge, this is the first report to characterize the epidemiology and genome, the evolutionary relationship, a possible cellular receptor, and pathohistological changes for this novel CoV, which causes diarrhea in ruminants. The subgenus *Embecovirus* includes members of *Betacoronavirus* 1, HCoV HKU1, RbCoV HKU14, and ChRCoV HKU24. CpCoV shares at least 90% aa sequence identity with all *Embecovirus* strains, supporting the suggestion that cpCoV belongs to the subgroup *Embecovirus*. Phylogenetically, cpCoV is positioned at the root of *Betacoronavirus* 1, and its location is distinct from that of other representative *Betacoronavirus* 1 strains. The genome of cpCoV also possesses features distinct from those of other *Embecovirus* strains, including a unique NS4a-4b protein and an evolutionarily separate S gene. The results reveal cpCoV should be a novel *Betacoronavirus* 1*.*

According to our phylogenetic analysis, cpCoV is more similar to HKU23 than BCoV and other BCoV-like viruses, as evidenced by the phylogenetic relatedness of most of the proteins predicted from the complete genome, and the RdRp, S, and N genes (Fig 3A–3D). The novel virus also shows a close relationship with *Oryx leucoryx* coronavirus, which might be a HKU23-associated virus and is most similar to HKU23 in phylogenetic analysis. CpCoV and dromedary HKU23 likely share a common ancestor, and a spillover event might have occurred between ruminants, cattle, camels, and wild animals such as *Oryx leucoryx*. The recent detection of the virus in goat herds and our molecular findings support the theory that goat is the natural host for cpCoV, though whether this virus is only spread among goats or if it has adapted to other ruminant hosts is still unknown, and further studies are needed including expanding the sampling scope and alignment of more CoV sequences from ruminants.

The global BCoV reference strains are primarily divided into GIa, GⅠb, GⅡa, and GⅡb [27]. Recently, surveillance of BCoV in China showed that the S gene of BCoV strains phylogenetically cluster into GⅡb. The consistency in the results of bootscanning and phylogenetic analyses also supports the possibility that cross-species recombination occurred in the S gene. Recombination is a common phenomenon in coronaviruses and is thought to contribute to the emergence of new pathotypes [45,47–49]. Our study has provided evidence for potential recombination events in the S and N gene regions of cpCoV and DcCoV (Fig 3E). Importantly, BCoV represents an excellent example of a CoV that has extensively crossed interspecies barriers, as several bovine-like CoVs have been identified as aetiologic pathogens of enteric and/or respiratory diseases in a diverse spectrum of ruminant species. Our sample collection sites were a long way from the nearest cattle farms, and the probability of BCoV transmission directly from cattle to goats was low. In addition, the discovery of cpCoVs with similar genomic characteristics from different regions of China confirmed that the cpCoVs had been circulating in goats for a long time. Based on a comparison of BCoV sequences in the database, the cpCoVs had not been detected or isolated from cattle until now. The S gene of BCoV-GX-NN190313 showed a close relationship to the cpCoVs; however, it was hard to assess whether this BCoV shared the same genomic characteristics with cpCoVs due to the absence of whole-genome information, especially data on the NS4a and NS4b genes. Phylogenetic analysis and recombination event evidences suggest that coinfection by different coronaviruses has occurred in ruminants of goats, cattle, camel, and other wild animals, which is possible as these ruminant animals inhabit the same geographic region, and the viruses potentially circulate among these populations.

Our findings offer new insights into the evolutionary origins of CoVs, with the evidence indicating interspecies transmission events have occurred. Based on the evolutionary trees, the cpCoVs are most closely related to DcCoV-HKU23 (Fig 3A–3D), which shares the same camel host as MERS, and possible recombination events are discovered between cpCoV and HKU23 (Fig 3E). Goats, camels, and *Oryx leucoryx* are ruminants, and more and more evidence is being found suggesting that ruminants are important intermediate hosts in the transmission of bat-borne or closely related CoVs from natural animals to human beings. For example, MERS-CoV might have been transmitted from bats to dromedary camels [14–17]. The 229E-related CoVs discovered in dromedary camels showed a close relationship to HCoV-229E [18–20]. HCoV-OC43 is believed to have originated from BCoVs and to have a close relationship with BCoV, and is presumed to have crossed species from cattle to humans around 1890 [50,51]. The high diversity of OC43-related *Betacoronavirus* 1 strains in livestock species supports the potential role of domestic ruminants as zoonotic sources [20]. *Embecovirus* members include many closely related mammalian CoVs, meaning that the threshold for transmission across mammalian species is low. The evolutionary history of these viruses is complex, and the ancestral origin of *Betacoronavirus* 1 remains obscure [25,44]. Our results suggest that goats are important hosts for *Embecovirus* and may harbor ancestral viruses belonging to *Betacoronavirus* 1 (Fig 3A–3D). The newly identified cpCoV might occupy an important position in the HCoV-OC43 evolutionary chain. CpCoV possesses a unique genome organization, especially regarding NS4a-4b, due to deletion mutations, and the NS4a sequence has the same number of amino acids as HCoV-OC43 and HKU23 (Fig 2A). Similar deletion events are discovered in regions of the S and NS5 genes of OrCoV/ISR1127. When compared with homologous genes of DcCoV-HKU23, 16 bp and 35 bp deletions are observed respectively, resulting in two distinct ORFs encoding 7.8-kDa and 4.7-kDa proteins [43]. Additionally, the sequences of NS4a and 4b in BCoV, DcCoV-HKU23, cpCoV, cpCoV/XJCJ2212, RbCoV-HKU14, and HCoV-OC43 show obvious stepwise deletions. Genetic mutations in CoVs may allow for the acquisition and maintenance of genes encoding diverse proteins and promote virus adaptation to specific hosts. Whether the NS4a-4b protein in particular contributed to the adaptation of the virus to a specific host is worthy of further study. Thus, there are reasons to suppose that inter-species transmission events have occurred in goats, camels, and other ruminant animals, and goats might play an important role as intermediate hosts that enable viral spread from natural hosts to humans. Nevertheless, further research is needed to fully elucidate the origins, evolution, cross-species transmission, and zoonotic potential of CoVs in animals. Reconstructing the evolutionary relationships of closely related viruses with specificities for different hosts may help elucidate the occurrence of virus strain emergence due to interspecies transmission events.

The cpCoV strains showed narrow cell tropism *in vitro* and susceptibility to human- and bovine-derived cell lines. CoVs are notoriously difficult to culture in cell lines. HCoV OC43 and HCoV229E induce only subtle or nonexistent CPEs after infection. In the present study, cpCoV is able to replicate in both MDBK and human epithelial (HRT-18G, HT-29) cells. HCoV OC43, BCoV, RbCoV HKU14, and ECoV are also known to replicate in HRT-18 cells, suggesting that these *Betacoronavirus* 1 share similar cellular tropisms. Cells expressing human-origin APN and ACE2 did not support the growth of cpCoV. Whether the most recent common ancestor of the closely related viruses cpCoV, BCoV, HKU23, and HCoV-OC43 originated from a virus that replicated in a single specific host and was then transmitted to the other two species, or even replicated in another species, cannot be inferred from the present data.

The causes of goat diarrhea are complex, with peste des petits ruminant virus, bovine viral diarrhea virus, border disease virus, rotavirus, and *Escherichia coli* among the possible pathogens leading to enteric disorder. The samples in our study were all sent for testing, and these pathogens were detected with variable positive rates. Coronaviruses occupied the highest positive rate with 16.7% (196/1172), and this rate reached 80% (24/30) in a farm with an outbreak of diarrhea. In animal experiments, both goats and cattle were susceptible to the cpCoV by intranasal infection and developed severe diarrhea, suggesting cpCoV is an important pathogen responsible for enteric disease in goats and cattle. In addition, cpCoV-infected goats showed mild respiratory symptoms such as nasal discharge and histopathologic lesions (Fig 4D and 4K). Evidence of virus isolation from bronchoalveolar lavage fluids and upper and lower respiratory tissues indicates BCoV challenge can result in respiratory infections [52]. CpCoV is associated with respiratory disease in goats according to our animal experiments. Moreover, the virus mainly replicated in the digestive tract and was shed through nasal secretions, saliva, and feces. The fecal–oral route should therefore be considered an important transmission route for the virus via which it may be transmitted to other animals. Therefore, risk analyses are particularly important for large herds of farm animals that are in direct contact with other animals and humans. The findings of this study extend the known host range of cpCoV to cattle, which provide a suitable model for the isolation and propagation of novel viruses, and indicates the potential for cross-species transmission and the substantial risk presented by cpCoV to cattle. Both goats and cattle can serve as model animals, but symptoms seem to be more severe in cpCoV-infected cattle. There are several possible reasons for this difference: cpCoV has never been detected or isolated from cattle in the field, potentially explaining why cattle are susceptible to this novel virus. The immune reaction varies between goats and cattle, with cattle being more susceptible to preventive and therapeutic interventions. CpCoV infections in calves could cause severe enteric disease, but whether the virus infects cattle naturally needs to be further investigated.

This is the first report of a previously undescribed virus, named cpCoV, that has characteristics distinct from those of known *Embecovirus* strains. This virus is highly prevalent and is thought to be one of the etiological agents behind recent outbreaks of enteric disease in goats. In addition, the virus induces severe diarrhea in cattle, suggesting there is a high risk of cross-species transmission during ruminant animal breeding. These findings highlight the importance of studying the phylogeny of CoVs to understand their evolutionary origins and cross-species transmission risk in ruminants. Extensive surveillance is required to define the epidemiology and evolution of cpCoVs in China and worldwide.

## Supporting information

S1 Fig IFA detection of anti-Spike protein monoclonal antibody for six representative cpCoV strains infecting HRT-18G, MDBK, and HT-29 cells. BCoV NXWZ2310 was used as positive control and showed positive results, and the mock infected cells showed negative results.
(TIF)

S2 Fig Identification of receptor usage of cpCoV. (A–C) ACE2 protein expression in 293T cells was identified by IFA with antibody against human ACE2. (D) SARS-CoV-2 pseudoviruses were used in the positive controls. (E–P) Live cpCoV infection of 293T-ACE2 showed human ACE2 expression did not enhance viral infection or entry.
(TIF)

S3 Fig Identification of receptor usage of cpCoV. (A–C) Human APN protein expression in BHK21 cells was identified by IFA with antibody against hAPN. (D–O) Live cpCoV infection of BHK-21-APN showed human APN expression did not enhance vital infection or entry.
(TIF)

S4 Fig Alignment of the NS4a-4b gene of cpCoV to that of BCoV, Yak coronavirus, HKU23, and HKU14. The 5 nt deletion in NS4a, the 10 nt deletion in NS4b, and the 54 nt deletion in XJCJ2301G compared with homologous regions in BCoV are marked in red. The start codon of the NS4a-4b gene is outlined in green, and the stop codon is outlined in orange. The nucleotide insertion compared with BCoV is outlined in blue.
(TIF)

S1 Table List of primers used in this study. (DOCX)

S2 Table Coding potential and predicted domains of the nonstructural proteins (nsp) of caprine coronavirus. (DOCX)

S3 Table Data for Fig 1G: Replication of cpCoV/AHFY2302G in HRT-18G, HT-29 and MDBK cells. (DOCX)

S4 Table Data for Fig 4E: Diarrhea index was evaluated in the CC-Goat and NC-Goat groups. (DOCX)

S5 Table Data for Fig 4F: CpCoV viral RNA shedding was detected in rectal swabs of goats. (DOCX)

S6 Table Data for Fig 4G: CpCoV viral RNA shedding was detected in nasal swabs of goats. (DOCX)

S7 Table Data for Fig 4H: CpCoV viral RNA shedding was detected in throat swabs of goats. (DOCX)

S8 Table Data for Fig 4I: The viral RNA load detected in organs of goats. (DOCX)

S9 Table Data for Fig 5A: Diarrhea index was evaluated in four groups of calves. (DOCX)

S10 Table Data for Fig 5B: The rectal temperature of calves in four groups. (DOCX)

S11 Table Data for Fig 5C: CpCoV viral RNA shedding was detected in rectal swabs of calves. (DOCX)

S12 Table Data for Fig 5D: CpCoV viral RNA shedding was detected in nasal swabs of calves. (DOCX)

S13 Table Data for Fig 5E: CpCoV viral RNA shedding was detected in throat swabs of calves. (DOCX)

S14 Table Data for Fig 5F: The viral RNA load detected in organs of calves. (DOCX)
