# Supplementary figures and images for: Discovery of a novel Betacoronavirus 1, cpCoV, in goats in China: The new risk of cross-species transmission
Source: PLoS Pathog. 2025 Mar 18;21(3):e1012974. doi: 10.1371/journal.ppat.1012974 (PMC11918373; doi:10.1371/journal.ppat.1012974)

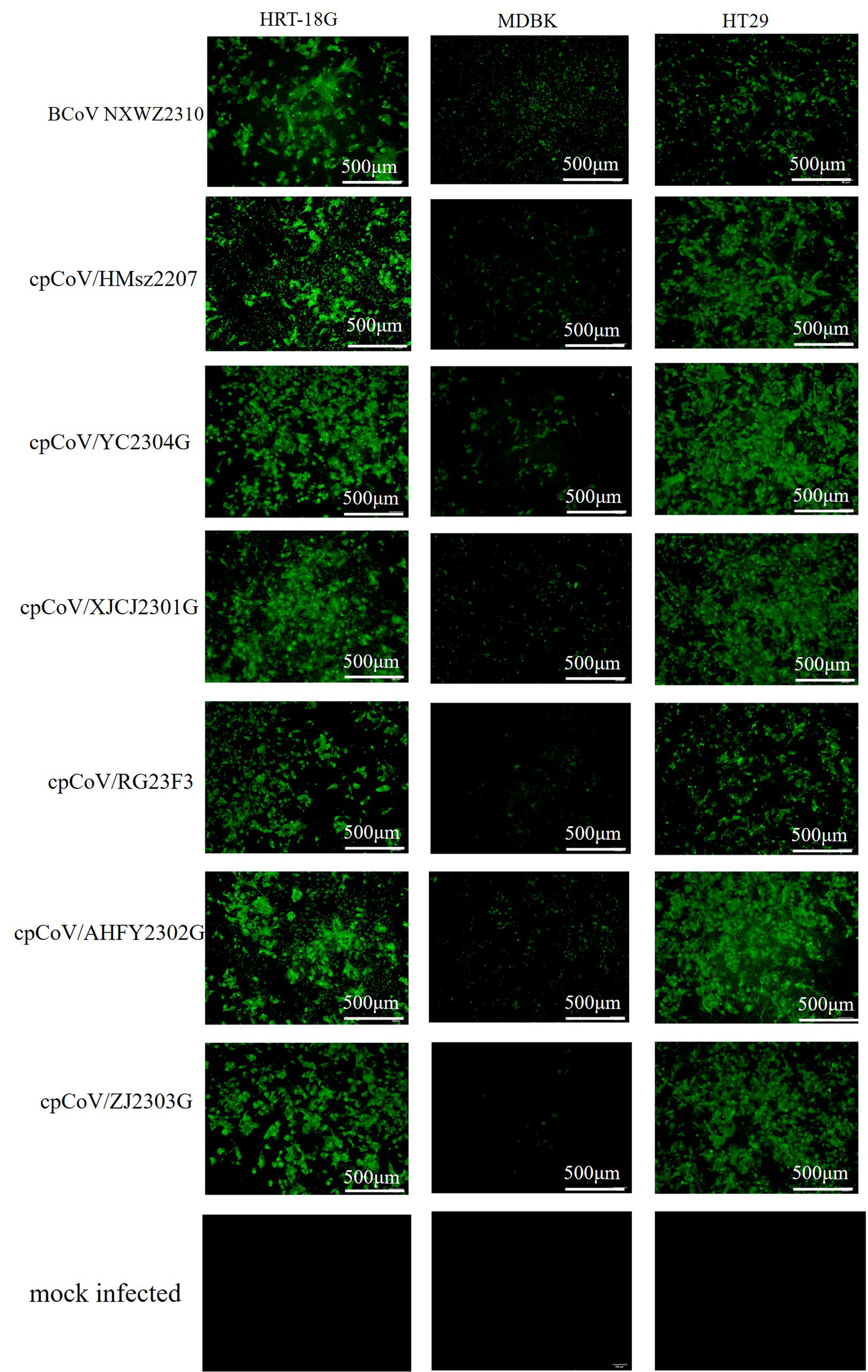

Supplement: S1 Fig — BCoV NXWZ2310 was used as positive control and showed positive results, and the mock infected cells showed negative results. (TIF) [file ppat.1012974.s001.tif]

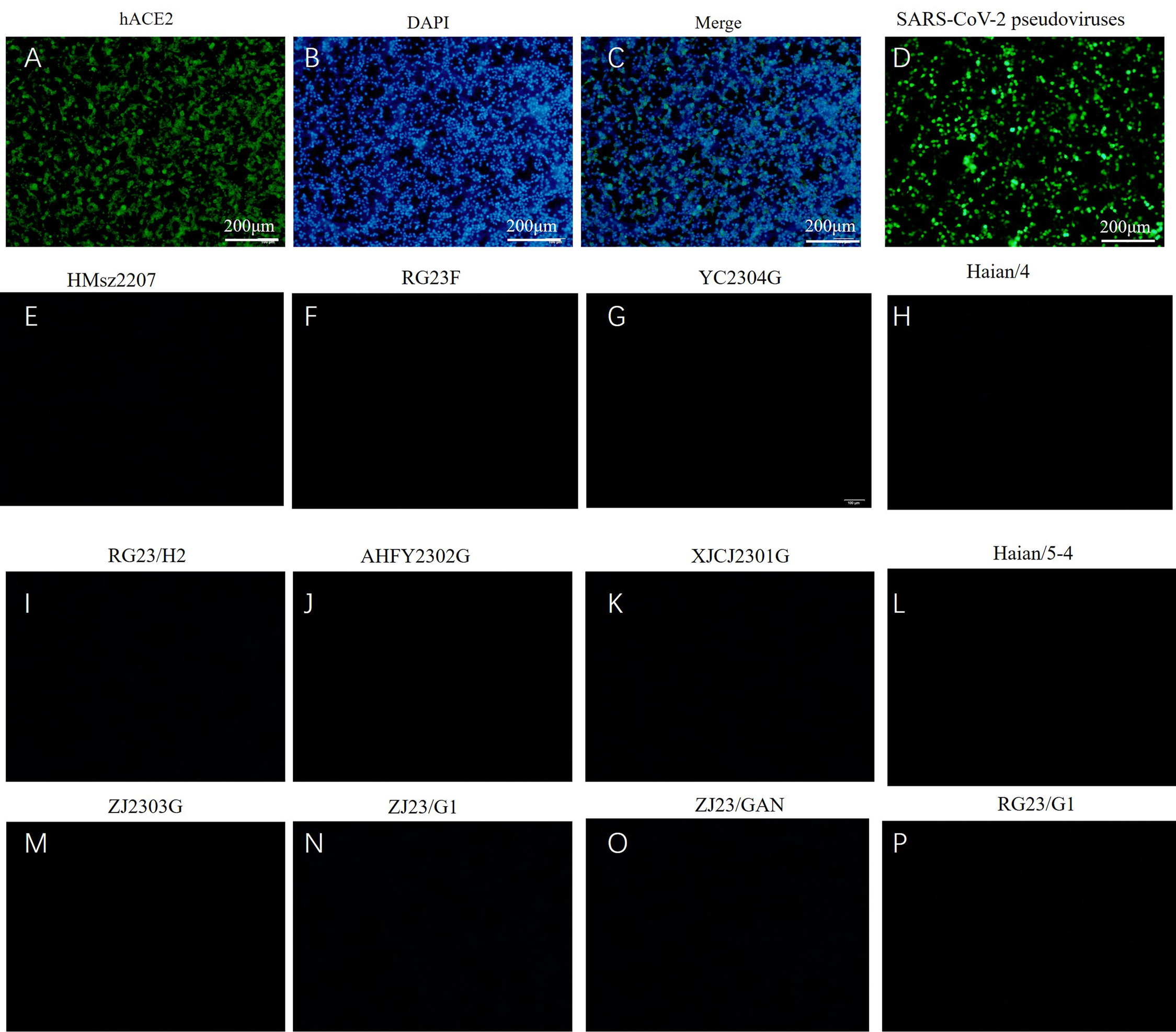

Supplement: S2 Fig — (A–C) ACE2 protein expression in 293T cells was identified by IFA with antibody against human ACE2. (D) SARS-CoV-2 pseudoviruses were used in the positive controls. (E–P) Live cpCoV infection of 293T-ACE2 showed human ACE2 expression did not enhance viral infection or entry. (TIF) [file ppat.1012974.s002.tif]

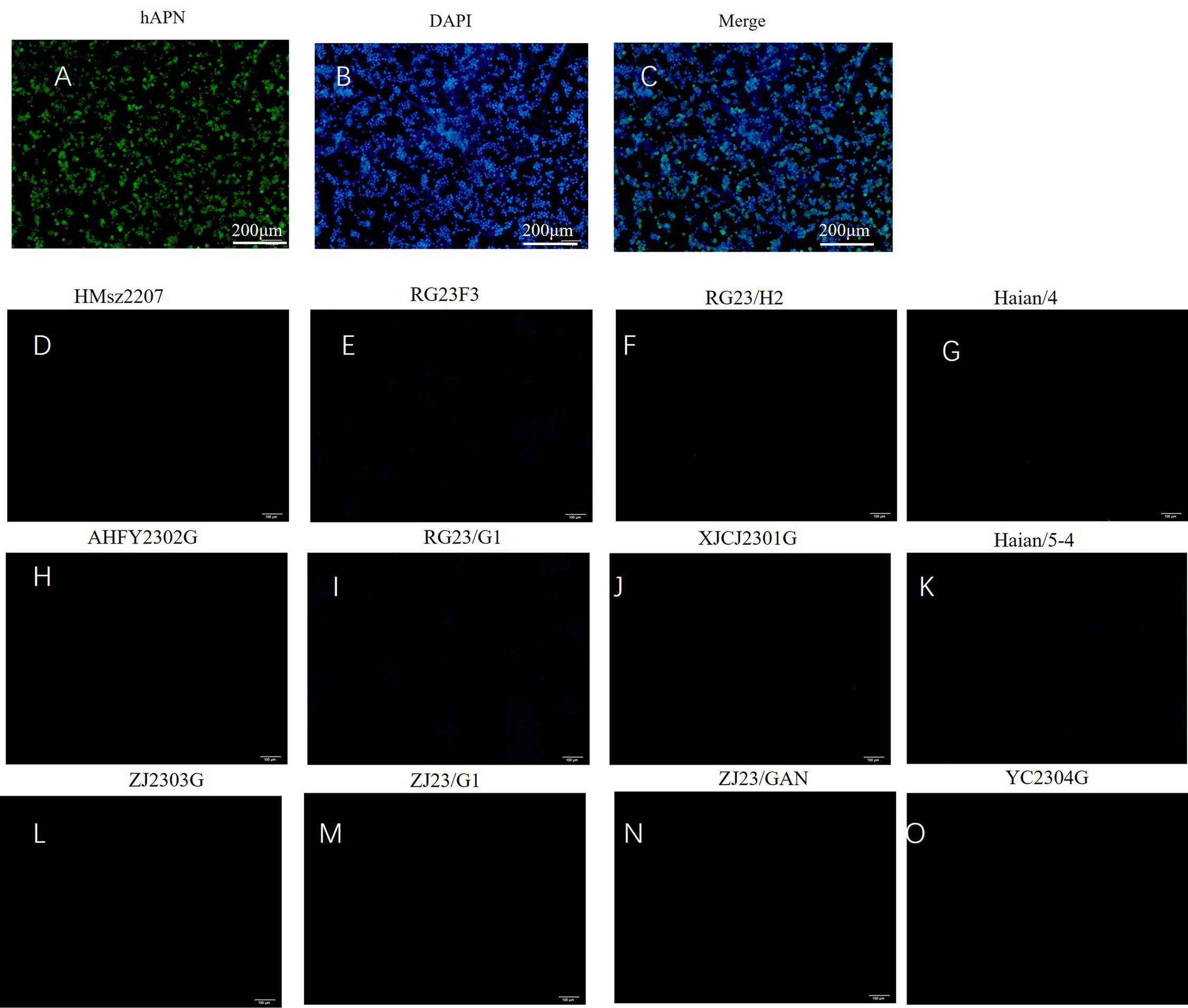

Supplement: S3 Fig — (A–C) Human APN protein expression in BHK21 cells was identified by IFA with antibody against hAPN. (D–O) Live cpCoV infection of BHK-21-APN showed human APN expression did not enhance vital infection or entry. (TIF) [file ppat.1012974.s003.tif]

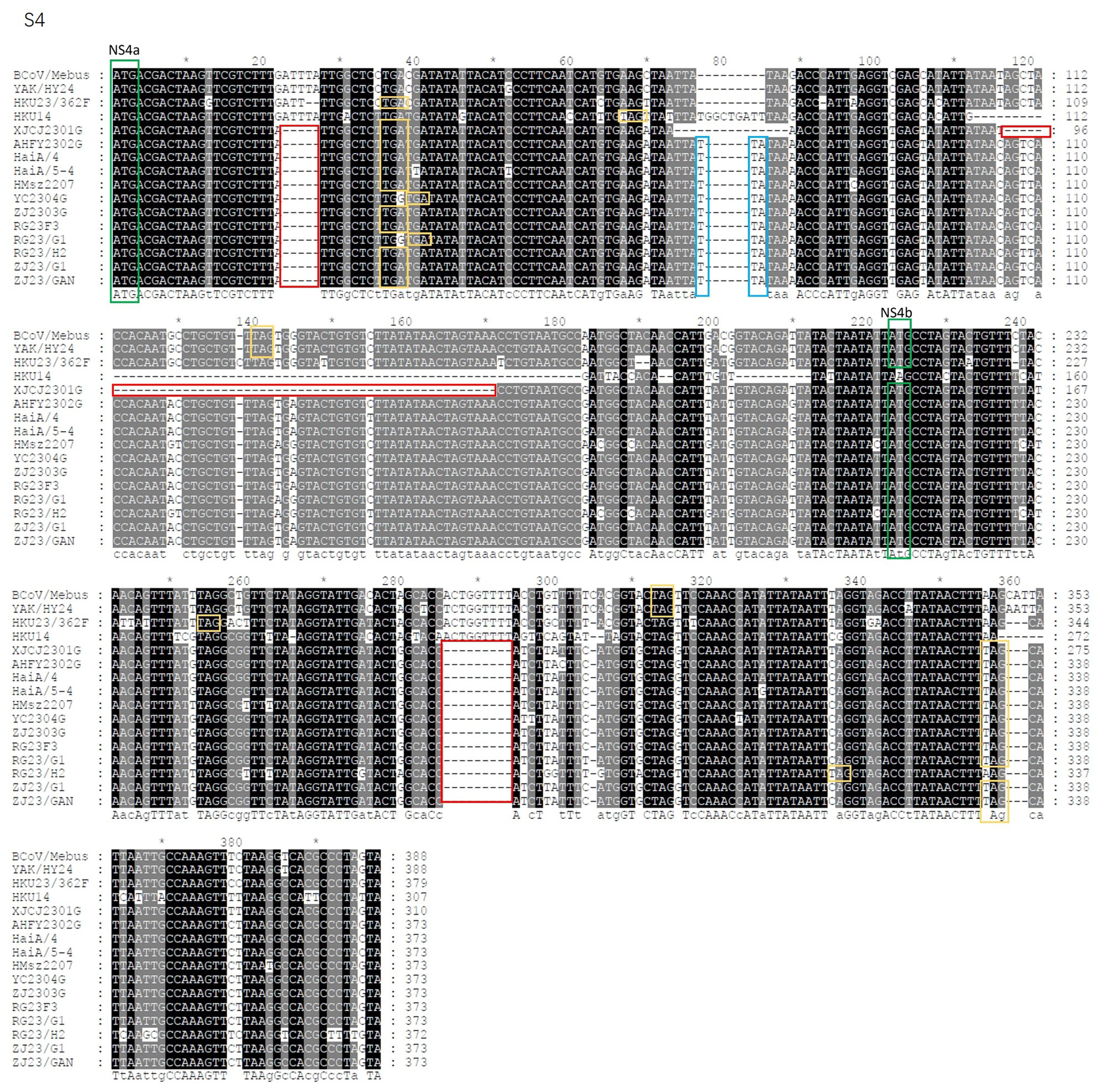

Supplement: S4 Fig — The 5 nt deletion in NS4a, the 10 nt deletion in NS4b, and the 54 nt deletion in XJCJ2301G compared with homologous regions in BCoV are marked in red. The start codon of the NS4a-4b gene is outlined in green, and the stop codon is outlined in orange. The nucleotide insertion compared with BCoV is outlined in blue. (TIF) [file ppat.1012974.s004.tif]
