# Supplementary material for: Discovery of a novel Betacoronavirus 1, cpCoV, in goats in China: The new risk of cross-species transmission
Source: PLoS Pathog. 2025 Mar 18;21(3):e1012974. doi: 10.1371/journal.ppat.1012974 (PMC11918373; doi:10.1371/journal.ppat.1012974)
Supplement: S1 Table — (DOCX) [file ppat.1012974.s005.docx]

S1_Table List of primers used in this study

S1_Table Primers sequences used in this study

| Primers | Primer sequence (5'－3') | Length/bp |
| --- | --- | --- |
| cpCoV-S1-F | TGCTGTTATAGGAGATTTAAAGT | 1403 |
| cpCoV-S1-R | GCTTTAAAACAATGTTGTGCATA |  |
| cpCoV-S2-F | GCTGCTAATGTTTCTGTTAGC | 1517 |
| cpCoV-S2-R | TCCAGTGCAATTATTATAAGCC |  |
| cpCoV-S3-F | GATCTGCTATAGAGGATTTAC | 1330 |
| cpCoV-S3-R | TAATTACTAACTCCTGGTGTC |  |
| cpCoV-NS4ab-F | TCTTCATATGCTGTTGTACAGG | 1038 |
| cpCoV-NS4ab-R | TCCACATCAAGAACTGGTGGT |  |
| BCoV-qF | TGCTGCCACGATGGTATTTTT | 101 |
| BCoV-qR | TGGTTACTAGCGACCCAGAAGAC |  |
| BCoV-q-probe | FAM-CTATCTTGGAACAGGACCGCATGCCA-BHQ1 |  |
| CoV-N-F | AAGGTGTGCCTATTGCACCAG | 500 |
| CoV-N-R | GCTTAGTTACTTGCTGTGGC |  |
