# Supplementary material for: Discovery of a novel Betacoronavirus 1, cpCoV, in goats in China: The new risk of cross-species transmission
Source: PLoS Pathog. 2025 Mar 18;21(3):e1012974. doi: 10.1371/journal.ppat.1012974 (PMC11918373; doi:10.1371/journal.ppat.1012974)
Supplement: S2 Table — (DOCX) [file ppat.1012974.s006.docx]

S2_Table Coding potential and predicted domains of the nonstructural proteins (nsp) of caprine coronavirus

|  | Protein position  Start end | | length | Cleavage site between nsp | Putative function or domain |
| --- | --- | --- | --- | --- | --- |
| orf1 | 1 | 7094 | 7094 |  |  |
| nsp1 | 1 | 246 | 246 | G/V | unknown |
| nsp2 | 247 | 851 | 605 | A/G | unknown |
| nsp3 | 852 | 2750 | 1899 | G/A | Acidic domain, hydrophobic  domain, ADRP, putative PL^pro^  domain PL1^pro^, PL2^pro^ |
| nsp4 | 2751 | 3246 | 496 | Q/S | Hydrophobic domain |
| nsp5 | 3247 | 3549 | 303 | Q/S | 3CL^pro^ |
| nsp6 | 3550 | 3836 | 287 | Q/S | Hydrophobic domain |
| nsp7 | 3837 | 3925 | 89 | Q/A | Unknown |
| nsp8 | 3926 | 4122 | 197 | Q/N | Unknown |
| nsp9 | 4123 | 4232 | 110 | Q/A | Unknown |
| nsp10 | 4233 | 4369 | 137 | Q/S | Unknown |
| nsp11 | 4370 | 4383 | 14 | S/V | Unknown |
| nsp12 | 4370 | 5297 | 928 | Q/S | RdRp |
| nsp13 | 5298 | 5900 | 603 | Q/C | Hel |
| nsp14 | 5901 | 6421 | 521 | Q/S | ExoN, N7-MTase |
| nsp15 | 6422 | 6795 | 374 | Q/A | NendoU |
| nsp16 | 6796 | 7094 | 299 |  | O-MT |
