# Supplementary material for: Discovery of a novel Betacoronavirus 1, cpCoV, in goats in China: The new risk of cross-species transmission
Source: PLoS Pathog. 2025 Mar 18;21(3):e1012974. doi: 10.1371/journal.ppat.1012974 (PMC11918373; doi:10.1371/journal.ppat.1012974)
Supplement: S3 Table — (DOCX) [file ppat.1012974.s007.docx]

S3_Table Data for Fig 1G: Replication of cpCoV/AHFY2302G in HRT-18G, HT-29 and MDBK cells (RNA copy number/mL)

|  | MDBK | | | HRT-18G | | | HT-29 | | | mock | | |
| --- | --- | --- | --- | --- | --- | --- | --- | --- | --- | --- | --- | --- |
| 0h | 1.35×10^2^ | 68 | 1.19×10^2^ | 56 | 2.09×10^2^ | 1.53×10^2^ | 1.05×10^2^ | 36 | 64 | / | / | / |
| 12h | 1.45×10^3^ | 7.76×10^2^ | 1.20×10^3^ | 7.81×10^3^ | 1.65×10^4^ | 5.04×10^3^ | 8.84×10^3^ | 1.99×10^4^ | 5.04×10^3^ | / | / | / |
| 24h | 8.84×10^3^ | 4.18×10^3^ | 3.46×10^3^ | 1.88×10^5^ | 8.36×10^4^ | 3.29×10^5^ | 5.07×10^4^ | 7.85×10^4^ | 1.01×10^5^ | / | / | / |
| 36h | 6.51×10^4^ | 4.77×10^4^ | 2.72×10^4^ | 1.32×10^8^ | 6.22×10^7^ | 8.50×10^7^ | 8.45×10^6^ | 3.53×10^6^ | 4.82×10^6^ | / | / | / |

/：undetected.
