# Supplementary material for: Discovery of a novel Betacoronavirus 1, cpCoV, in goats in China: The new risk of cross-species transmission
Source: PLoS Pathog. 2025 Mar 18;21(3):e1012974. doi: 10.1371/journal.ppat.1012974 (PMC11918373; doi:10.1371/journal.ppat.1012974)
Supplement: S4 Table — (DOCX) [file ppat.1012974.s008.docx]

S4_Table Data for Fig 4E: Diarrhea index was evaluated in the CC-Goat and NC-Goat groups

| dpi | NC-Goat | | | CC-Goat | | |
| --- | --- | --- | --- | --- | --- | --- |
| 0 | 0 | 0 | 0 | 0 | 0 | 0 |
| 1 | 0 | 0 | 0 | 0 | 0 | 0 |
| 2 | 0 | 0 | 0 | 1 | 0 | 0 |
| 3 | 0 | 0 | 0 | 2 | 1 | 2 |
| 4 | 0 | 0 | 0 | 3 | 2 | 2.5 |
| 5 | 0 | 0 | 0 | 2 | 3 | 2.5 |
| 6 | 0 | 0 | 0 | 2 | 2 | 2 |
| 7 | 0 | 0 | 0 | 1 | 1 | 1.5 |
| 8 | 0 | 0 | 0 | 0 | 0 | 1 |
| 9 | 0 | 0 | 0 | 0 | 0 | 0 |
| 10 | 0 | 0 | 0 | 0 | 0 | 0 |
