# Supplementary material for: Discovery of a novel Betacoronavirus 1, cpCoV, in goats in China: The new risk of cross-species transmission
Source: PLoS Pathog. 2025 Mar 18;21(3):e1012974. doi: 10.1371/journal.ppat.1012974 (PMC11918373; doi:10.1371/journal.ppat.1012974)
Supplement: S5 Table — (DOCX) [file ppat.1012974.s009.docx]

S5_Table Data for Fig 4F: CpCoV viral RNA shedding was detected in rectal swabs of goats (RNA copy number/mL)

| dpi | NC-Goat | | | CC-Goat | | |
| --- | --- | --- | --- | --- | --- | --- |
| 0 | / | / | / | / | / | / |
| 1 | / | / | / | 1.69×10^2^ | 2.64×10^2^ | 32 |
| 2 | / | / | / | 3.06×10^5^ | 1.06×10^5^ | 6.88×10^5^ |
| 3 | / | / | / | 9.98×10^7^ | 1.16×10^8^ | 1.78×10^7^ |
| 4 | / | / | / | 1.45×10^6^ | 1.56×10^7^ | 1.13×10^7^ |
| 5 | / | / | / | 5.05×10^8^ | 5.68×10^7^ | 2.95×10^8^ |
| 6 | / | / | / | 1.51×10^7^ | 9.69×10^6^ | 5.74×10^7^ |
| 7 | / | / | / | 1.99×10^7^ | 7.10×10^7^ | 7.91×10^7^ |
| 8 | / | / | / | 1.98×10^5^ | 3.31×10^5^ | 7.05×10^4^ |
| 9 | / | / | / | 4.05×10^3^ | 5.25×10^4^ | 3.74×10^4^ |
| 10 | / | / | / | 1.83×10^2^ | 7.25×10^2^ | 2.84×10^2^ |
| 11 | / | / | / | 63 | 1.15×10^2^ | 35 |

/：undetected.
