# Supplementary material for: Discovery of a novel Betacoronavirus 1, cpCoV, in goats in China: The new risk of cross-species transmission
Source: PLoS Pathog. 2025 Mar 18;21(3):e1012974. doi: 10.1371/journal.ppat.1012974 (PMC11918373; doi:10.1371/journal.ppat.1012974)
Supplement: S6 Table — (DOCX) [file ppat.1012974.s010.docx]

S6_Table Data for Fig 4G: CpCoV viral RNA shedding was detected in nasal swabs of goats (RNA copy number/mL)

| dpi | NC-Goat | | | CC-Goat | | |
| --- | --- | --- | --- | --- | --- | --- |
| 0 | / | / | / | / | / | / |
| 1 | / | / | / | 51 | 43 | 17 |
| 2 | / | / | / | 100 | 200 | 120 |
| 3 | / | / | / | 8.05×10^3^ | 924 | 1.03×10^4^ |
| 4 | / | / | / | 8.73×10^3^ | 4.77×10^4^ | 9.24×10^4^ |
| 5 | / | / | / | 8.72×10^5^ | 4.47×10^6^ | 2.01×10^6^ |
| 6 | / | / | / | 7.90×10^4^ | 1.0×10^4^ | 1.63×10^5^ |
| 7 | / | / | / | 4.03×10^4^ | 2.13×10^5^ | 5.11×10^4^ |
| 8 | / | / | / | 5.10×10^4^ | 2.44×10^4^ | 1.49×10^5^ |
| 9 | / | / | / | 6.31×10^2^ | 4.36×10^3^ | 4.10×10^3^ |
| 10 | / | / | / | 1.19×10^2^ | 530 | 7.43×10^2^ |
| 11 | / | / | / | 59 | 110 | 206 |

/：undetected.
