# Supplementary material for: Discovery of a novel Betacoronavirus 1, cpCoV, in goats in China: The new risk of cross-species transmission
Source: PLoS Pathog. 2025 Mar 18;21(3):e1012974. doi: 10.1371/journal.ppat.1012974 (PMC11918373; doi:10.1371/journal.ppat.1012974)
Supplement: S7 Table — (DOCX) [file ppat.1012974.s011.docx]

S7_Table Data for Fig 4H: CpCoV viral RNA shedding was detected in throat swabs of goats (RNA copy number/mL)

| dpi | NC-Goat | | | CC-Goat | | |
| --- | --- | --- | --- | --- | --- | --- |
| 0 | / | / | / | / | / | / |
| 1 | / | / | / | 40 | 59 | 20 |
| 2 | / | / | / | 3.64×10^2^ | 1.33×10^3^ | 2.0×10^3^ |
| 3 | / | / | / | 2.08×10^3^ | 5.01×10^3^ | 1.59×10^4^ |
| 4 | / | / | / | 2.69×10^3^ | 6.01×10^3^ | 1.14×10^4^ |
| 5 | / | / | / | 3.08×10^3^ | 4.0×10^3^ | 1.57×10^4^ |
| 6 | / | / | / | 8.52×10^3^ | 7.57×10^3^ | 3.82×10^4^ |
| 7 | / | / | / | 1.07×10^2^ | 2.04×10^2^ | 5.87×10^2^ |
| 8 | / | / | / | 55 | 1.34×10^2^ | 2.07×10^2^ |
| 9 | / | / | / | 1.28×10^2^ | 82 | 2.57×10^2^ |
| 10 | / | / | / | 1.11×10^2^ | 51 | 85 |
| 11 | / | / | / | 61 | 42 | 30 |

/：undetected.
