# Supplementary material for: Discovery of a novel Betacoronavirus 1, cpCoV, in goats in China: The new risk of cross-species transmission
Source: PLoS Pathog. 2025 Mar 18;21(3):e1012974. doi: 10.1371/journal.ppat.1012974 (PMC11918373; doi:10.1371/journal.ppat.1012974)
Supplement: S8 Table — (DOCX) [file ppat.1012974.s012.docx]

S8_Table Data for Fig 4I: The viral RNA load detected in organs of goats (RNA copy number/mL)

|  | NC-Goat | | | CC-Goat | | |
| --- | --- | --- | --- | --- | --- | --- |
| heart | / | / | / | 1.27×10^4^ | 3.26×10^3^ | 2.47×10^3^ |
| liver | / | / | / | 1.37×10^3^ | 1.20×10^3^ | 1.91×10^3^ |
| spleen | / | / | / | 17 | 1.31×10^4^ | 20 |
| lung | / | / | / | 2.09×10^3^ | 3.83×10^4^ | 1.02×10^3^ |
| renal | / | / | / | 1.40×10^4^ | 180 | 476 |
| trachea | / | / | / | 519 | 2.86×10^4^ | 424 |
| lymph nodes | / | / | / | 788 | 5.05×10^3^ | 3.16×10^3^ |
| duodenum | / | / | / | 766 | 6.80×10^3^ | 1.56×10^3^ |
| jejunum | / | / | / | 1.03×10^4^ | 510 | 5.11×10^3^ |
| ileum | / | / | / | 20 | 8.80×10^3^ | 7.09×10^4^ |
| cecum | / | / | / | 3.92×10^6^ | 2.94×10^5^ | 2.26×10^5^ |
| colon | / | / | / | 2.20×10^5^ | 1.34×10^4^ | 8.80×10^4^ |
| rectum | / | / | / | 2.83×10^5^ | 7.00×10^4^ | 1.63×10^5^ |

/：undetected.
