# Supplementary material for: Discovery of a novel Betacoronavirus 1, cpCoV, in goats in China: The new risk of cross-species transmission
Source: PLoS Pathog. 2025 Mar 18;21(3):e1012974. doi: 10.1371/journal.ppat.1012974 (PMC11918373; doi:10.1371/journal.ppat.1012974)
Supplement: S9 Table — (DOCX) [file ppat.1012974.s013.docx]

S9_Table Data for Fig 5A: Diarrhea index was evaluated in four groups of calves

| Diarrhea index of calves | | | | | | | | | | | | |
| --- | --- | --- | --- | --- | --- | --- | --- | --- | --- | --- | --- | --- |
| dpi | NC-7 | | | NC-14 | | | CC-7 | | | CC-14 | | |
| 0 | 0 | 0 | 0 | 0 | 0 | 0 | 0 | 0 | 0 | 0 | 0 | 0 |
| 1 | 0 | 0 | 0 | 0 | 0 | 0 | 1 | 1 | 0 | 0 | 1 | 0 |
| 2 | 0 | 0 | 0 | 0 | 0 | 0 | 2 | 2 | 0.5 | 1 | 1.5 | 0 |
| 3 | 0 | 0 | 0 | 0 | 0 | 0 | 3 | 3 | 2 | 2 | 1.5 | 2 |
| 4 | 0 | 0 | 0 | 0 | 0 | 0 | 2.5 | 2 | 2 | 2 | 2 | 3 |
| 5 | 0 | 0 | 0 | 0 | 0 | 0 | 2 | 1 | 2.5 | 2 | 2 | 3 |
| 6 | 0 | 0 | 0 | 0 | 0 | 0 | 2 | 3 | 2 | 2 | 2 | 3 |
| 7 | 0 | 0 | 0 | 0 | 0 | 0 | 3 | 3 | 2.5 | 2.5 | 3 | 3 |
| 8 |  |  |  | 0 | 0 | 0 |  |  |  | 2 | 0 | 2 |
| 9 |  |  |  | 0 | 0 | 0 |  |  |  | 1.5 | 0 | 2 |
| 10 |  |  |  | 0 | 0 | 0 |  |  |  | 1 | 0 | 1 |
| 11 |  |  |  | 0 | 0 | 0 |  |  |  | 1 | 0 | 0 |
| 12 |  |  |  | 0 | 0 | 0 |  |  |  | 0 | 0 | 0 |
| 13 |  |  |  | 0 | 0 | 0 |  |  |  | 0 | 0 | 0 |
| 14 |  |  |  | 0 | 0 | 0 |  |  |  | 0 | 0 | 0 |
