# Supplementary material for: Discovery of a novel Betacoronavirus 1, cpCoV, in goats in China: The new risk of cross-species transmission
Source: PLoS Pathog. 2025 Mar 18;21(3):e1012974. doi: 10.1371/journal.ppat.1012974 (PMC11918373; doi:10.1371/journal.ppat.1012974)
Supplement: S10 Table — (DOCX) [file ppat.1012974.s014.docx]

S10_Table Data for Fig 5B: The rectal temperature of calves in four groups

| The rectal temperature from calves (℃) | | | | | | | | | | | | |
| --- | --- | --- | --- | --- | --- | --- | --- | --- | --- | --- | --- | --- |
| dpi | NC-7 | | | NC-14 | | | CC-7 | | | CC-14 | | |
| 0 | 38.3 | 39.0 | 38.0 | 39.0 | 38.5 | 38.0 | 38.5 | 39.0 | 38.0 | 38.6 | 39.2 | 38.0 |
| 1 | 38.1 | 38.3 | 38.7 | 39.1 | 38.2 | 38.9 | 38.3 | 38.0 | 38.1 | 39.1 | 39.8 | 39.2 |
| 2 | 38.5 | 38.2 | 39.0 | 38.3 | 38.6 | 39.0 | 38.5 | 39.0 | 39.0 | 39.8 | 39.2 | 38.7 |
| 3 | 38.5 | 38.5 | 39.0 | 39.0 | 39.0 | 39.0 | 39.0 | 39.3 | 38.5 | 40.1 | 38.9 | 38.7 |
| 4 | 38.9 | 38.4 | 38.5 | 38.7 | 39.0 | 38.5 | 40.1 | 39.7 | 38.9 | 40.5 | 38.9 | 39.0 |
| 5 | 38.9 | 38.7 | 38.7 | 38.6 | 38.9 | 39.1 | 40.3 | 39.8 | 38.9 | 40.1 | 39.4 | 39.8 |
| 6 | 39.1 | 39.0 | 38.7 | 38.8 | 39.0 | 38.7 | 40.0 | 39.6 | 39.1 | 41.0 | 39.1 | 39.5 |
| 7 | 38.6 | 39.2 | 38.5 | 39.0 | 39.2 | 38.0 | 40.6 | 40.1 | 39.0 | 40.4 | 39.0 | 39.4 |
| 8 |  |  |  | 39.0 | 38.2 | 39.0 |  |  |  | 39.9 | 39.2 | 39.2 |
| 9 |  |  |  | 38.7 | 38.4 | 39.0 |  |  |  | 39.8 | 39.1 | 38.8 |
| 10 |  |  |  | 38.1 | 38.2 | 39.0 |  |  |  | 39.5 | 38.9 | 38.7 |
| 11 |  |  |  | 38.5 | 38.5 | 38.7 |  |  |  | 39.3 | 39.1 | 39.0 |
| 12 |  |  |  | 39.0 | 38.7 | 38.1 |  |  |  | 38.9 | 38.9 | 39.0 |
| 13 |  |  |  | 38.8 | 39.0 | 38.5 |  |  |  | 38.7 | 39.0 | 38.8 |
| 14 |  |  |  | 38.5 | 38.7 | 38.8 |  |  |  | 38.8 | 39.0 | 39.1 |
