# Supplementary material for: Discovery of a novel Betacoronavirus 1, cpCoV, in goats in China: The new risk of cross-species transmission
Source: PLoS Pathog. 2025 Mar 18;21(3):e1012974. doi: 10.1371/journal.ppat.1012974 (PMC11918373; doi:10.1371/journal.ppat.1012974)
Supplement: S11 Table — (DOCX) [file ppat.1012974.s015.docx]

S11_Table Data for Fig 5C: CpCoV viral RNA shedding was detected in rectal swabs of calves (RNA copy number/mL)

| CpCoV viral RNA shedding was detected in rectal swabs of calves (RNA copy number/mL) | | | | | | | | | | | | |
| --- | --- | --- | --- | --- | --- | --- | --- | --- | --- | --- | --- | --- |
| dpi | NC-7 | | | NC-14 | | | CC-7 | | | CC-14 | | |
| 0 | / | / | / | / | / | / | / | / | / | / | / | / |
| 1 | / | / | / | / | / | / | 1.55×10^2^ | 5.28×10^2^ | 1.15×10^2^ | 1.46×10^2^ | 9.89×10^2^ | 1.34×10^3^ |
| 2 | / | / | / | / | / | / | 1.15×10^5^ | 2.00×10^4^ | 6.38×10^3^ | 2.51×10^4^ | 2.74×10^4^ | 6.43×10^4^ |
| 3 | / | / | / | / | / | / | 8.47×10^6^ | 1.09×10^6^ | 2.09×10^6^ | 3.09×10^6^ | 2.09×10^6^ | 2.09×10^6^ |
| 4 | / | / | / | / | / | / | 7.36×10^6^ | 1.37×10^6^ | 1.50×10^6^ | 5.37×10^5^ | 5.85×10^5^ | 1.15×10^5^ |
| 5 | / | / | / | / | / | / | 1.04×10^7^ | 9.38×10^6^ | 1.43×10^8^ | 3.15×10^7^ | 1.12×10^7^ | 8.38×10^7^ |
| 6 | / | / | / | / | / | / | 2.35×10^7^ | 4.76×10^7^ | 2.13×10^7^ | 1.90×10^7^ | 1.43×10^7^ | 8.20×10^7^ |
| 7 | / | / | / | / | / | / | 1.07×10^7^ | 1.38×10^7^ | 3.17×10^6^ | 8.31×10^6^ | 9.09×10^6^ | 3.05×10^7^ |
| 8 |  |  |  | / | / | / |  |  |  | 7.24×10^4^ | 3.93×10^5^ | 1.03×10^6^ |
| 9 |  |  |  | / | / | / |  |  |  | 1.29×10^5^ | 1.03×10^4^ | 4.74×10^4^ |
| 10 |  |  |  | / | / | / |  |  |  | 8.98×10^5^ | 1.13×10^5^ | 2.57×10^5^ |
| 11 |  |  |  | / | / | / |  |  |  | 1.32×10^6^ | 3.26×10^5^ | 2.57×10^5^ |
| 12 |  |  |  | / | / | / |  |  |  | 6.81×10^3^ | 2.13×10^3^ | 1.07×10^4^ |
| 13 |  |  |  | / | / | / |  |  |  | 6.84×10^2^ | 1.70×10^2^ | 9.77×10^2^ |
| 14 |  |  |  | / | / | / |  |  |  | 3.05×10^2^ | 2.39×10^2^ | 2.13×10^2^ |

/：undetected.
