# Supplementary material for: Discovery of a novel Betacoronavirus 1, cpCoV, in goats in China: The new risk of cross-species transmission
Source: PLoS Pathog. 2025 Mar 18;21(3):e1012974. doi: 10.1371/journal.ppat.1012974 (PMC11918373; doi:10.1371/journal.ppat.1012974)
Supplement: S12 Table — (DOCX) [file ppat.1012974.s016.docx]

S12_Table Data for Fig 5D: CpCoV viral RNA shedding was detected in nasal swabs of calves (RNA copy number/mL)

| CpCoV viral RNA shedding was detected in nasal swabs of calves (RNA copy number/mL) | | | | | | | | | | | | |
| --- | --- | --- | --- | --- | --- | --- | --- | --- | --- | --- | --- | --- |
| dpi | NC-7 | | | NC-14 | | | CC-7 | | | CC-14 | | |
| 0 | / | / | / | / | / | / | / | / | / | / | / | / |
| 1 | / | / | / | / | / | / | 2.48×10^5^ | 6.08×10^5^ | 2.74×10^4^ | 1.24×10^6^ | 4.27×10^5^ | 2.84×10^5^ |
| 2 | / | / | / | / | / | / | 2.13×10^6^ | 4.73×10^6^ | 8.24×10^6^ | 1.43×10^7^ | 5.53×10^6^ | 6.12×10^5^ |
| 3 | / | / | / | / | / | / | 5.11×10^6^ | 1.98×10^6^ | 1.30×10^7^ | 8.89×10^6^ | 4.93×10^5^ | 8.91×10^5^ |
| 4 | / | / | / | / | / | / | 2.38×10^6^ | 3.03×10^6^ | 4.56×10^6^ | 4.41×10^6^ | 6.22×10^5^ | 1.35×10^6^ |
| 5 | / | / | / | / | / | / | 8.48×10^6^ | 3.11×10^6^ | 1.20×10^7^ | 7.34×10^6^ | 9.15×10^5^ | 1.76×10^6^ |
| 6 | / | / | / | / | / | / | 9.45×10^5^ | 8.74×10^5^ | 6.57×10^5^ | 1.24×10^6^ | 2.45×10^5^ | 2.40×10^5^ |
| 7 | / | / | / | / | / | / | 1.15×10^6^ | 1.86×10^6^ | 8.07×10^5^ | 2.40×10^6^ | 9.89×10^5^ | 2.64×10^5^ |
| 8 |  |  |  | / | / | / |  |  |  | 3.46×10^5^ | 3.81×10^4^ | 1.50×10^5^ |
| 9 |  |  |  | / | / | / |  |  |  | 5.29×10^5^ | 1.93×10^5^ | 9.18×10^4^ |
| 10 |  |  |  | / | / | / |  |  |  | 5.80×10^4^ | 1.28×10^4^ | 1.91×10^4^ |
| 11 |  |  |  | / | / | / |  |  |  | 1.88×10^4^ | 8.01×10^3^ | 6.54×10^3^ |
| 12 |  |  |  | / | / | / |  |  |  | 2.86×10^4^ | 9.09×10^3^ | 4.93×10^4^ |
| 13 |  |  |  | / | / | / |  |  |  | 5.70×10^2^ | 5.10×10^2^ | 6.17×10^2^ |
| 14 |  |  |  | / | / | / |  |  |  | 4.80×10^2^ | 2.55×10^2^ | 7.31×10^2^ |

/：undetected.
