# Supplementary material for: Discovery of a novel Betacoronavirus 1, cpCoV, in goats in China: The new risk of cross-species transmission
Source: PLoS Pathog. 2025 Mar 18;21(3):e1012974. doi: 10.1371/journal.ppat.1012974 (PMC11918373; doi:10.1371/journal.ppat.1012974)
Supplement: S13 Table — (DOCX) [file ppat.1012974.s017.docx]

S13_Table Data for Fig 5E: CpCoV viral RNA shedding was detected in throat swabs of calves

| CpCoV viral RNA shedding was detected in throat swabs of calves (RNA copy number/mL) | | | | | | | | | | | | |
| --- | --- | --- | --- | --- | --- | --- | --- | --- | --- | --- | --- | --- |
| dpi | NC-7 | | | NC-14 | | | CC-7 | | | CC-14 | | |
| 0 | / | / | / | / | / | / | / | / | / | / | / | / |
| 1 | / | / | / | / | / | / | 4.74×10^3^ | 1.92×10^4^ | 1.47×10^3^ | 2.43×10^4^ | 1.96×10^3^ | 1.51×10^4^ |
| 2 | / | / | / | / | / | / | 5.25×10^5^ | 3.10×10^5^ | 7.48×10^5^ | 2.07×10^6^ | 9.31×10^5^ | 1.61×10^5^ |
| 3 | / | / | / | / | / | / | 1.96×10^6^ | 1.40×10^7^ | 9.12×10^6^ | 3.84×10^6^ | 1.97×10^6^ | 2.89×10^6^ |
| 4 | / | / | / | / | / | / | 1.75×10^5^ | 3.86×10^5^ | 4.26×10^5^ | 6.81×10^4^ | 9.61×10^4^ | 1.70×10^5^ |
| 5 | / | / | / | / | / | / | 1.28×10^5^ | 2.49×10^6^ | 1.62×10^5^ | 5.05×10^5^ | 2.04×10^5^ | 1.17×10^5^ |
| 6 | / | / | / | / | / | / | 2.90×10^5^ | 5.85×10^5^ | 4.88×10^4^ | 3.27×10^5^ | 9.41×10^4^ | 4.60×10^5^ |
| 7 | / | / | / | / | / | / | 1.95×10^5^ | 3.86×10^5^ | 5.93×10^4^ | 3.39×10^5^ | 9.96×10^4^ | 1.84×10^5^ |
| 8 |  |  |  | / | / | / |  |  |  | 7.82×10^4^ | 4.22×10^2^ | 3.92×10^4^ |
| 9 |  |  |  | / | / | / |  |  |  | 9.75×10^3^ | 1.37×10^3^ | 1.10×10^4^ |
| 10 |  |  |  | / | / | / |  |  |  | 8.88×10^3^ | 1.01×10^3^ | 4.27×10^3^ |
| 11 |  |  |  | / | / | / |  |  |  | 1.36×10^4^ | 6.37×10^3^ | 1.15×10^3^ |
| 12 |  |  |  | / | / | / |  |  |  | 6.38×10^3^ | 6.39×10^2^ | 1.18×10^3^ |
| 13 |  |  |  | / | / | / |  |  |  | 5.39×10^2^ | 3.10×10^2^ | 4.04×10^2^ |
| 14 |  |  |  | / | / | / |  |  |  | 8.46×10^3^ | 2.40×10^2^ | 3.43×10^2^ |

/：undetected.
