# Supplementary material for: Discovery of a novel Betacoronavirus 1, cpCoV, in goats in China: The new risk of cross-species transmission
Source: PLoS Pathog. 2025 Mar 18;21(3):e1012974. doi: 10.1371/journal.ppat.1012974 (PMC11918373; doi:10.1371/journal.ppat.1012974)
Supplement: S14 Table — (DOCX) [file ppat.1012974.s018.docx]

S14_Table Data for Fig 5F: The viral RNA load detected in organs of calves

| The viral RNA load detected in organs of calves (RNA copy number/mL) | | | | | | | | | | | | |
| --- | --- | --- | --- | --- | --- | --- | --- | --- | --- | --- | --- | --- |
|  | NC-7 | | | NC-14 | | | CC-7 | | | CC-14 | | |
| heart | / | / | / | / | / | / | 2.04×10^7^ | 2.65×10^5^ | 1.83×10^4^ | 4.24×10^2^ | 3.85×10^2^ | 8.02×10^2^ |
| liver | / | / | / | / | / | / | 1.54×10^6^ | 1.18×10^4^ | 8.34×10^2^ | 1.71×10^1^ | 7.01×10^2^ | 2.77×10^2^ |
| spleen | / | / | / | / | / | / | 1.83×10^6^ | 9.72×10^3^ | 4.01×10^3^ | 3.04×10^4^ | 1.40×10^3^ | 3.34×10^2^ |
| lung | / | / | / | / | / | / | 1.72×10^6^ | 7.27×10^4^ | 1.89×10^4^ | 4.62×10^2^ | 9.03×10^2^ | 1.18×10^2^ |
| renal | / | / | / | / | / | / | 1.00×10^6^ | 8.09×10^3^ | 1.71×10^1^ | 8.77×10^2^ | 2.54×10^3^ | 1.75×10^2^ |
| trachea | / | / | / | / | / | / | 2.16×10^6^ | 4.42×10^5^ | 5.21×10^2^ | 1.72×10^3^ | 1.11×10^2^ | 3.36×10^2^ |
| lymph nodes | / | / | / | / | / | / | 2.40×10^6^ | 8.01×10^5^ | 1.94×10^5^ | 5.10×10^4^ | 7.23×10^3^ | 3.31×10^4^ |
| duodenum | / | / | / | / | / | / | 1.54×10^7^ | 1.38×10^7^ | 1.20×10^4^ | 1.35×10^7^ | 5.42×10^1^ | 1.71×10^1^ |
| ileum | / | / | / | / | / | / | 1.72×10^8^ | 7.49×10^7^ | 3.83×10^3^ | 1.82×10^4^ | 7.83×10^3^ | 8.53×10^4^ |
| jejunum | / | / | / | / | / | / | 4.77×10^7^ | 1.04×10^8^ | 8.21×10^4^ | 7.37×10^4^ | 2.36×10^2^ | 5.04×10^3^ |
| cecum | / | / | / | / | / | / | 3.27×10^9^ | 3.89×10^6^ | 7.33×10^6^ | 3.13×10^3^ | 1.22×10^3^ | 1.62×10^3^ |
| colon | / | / | / | / | / | / | 7.81×10^8^ | 7.06×10^8^ | 8.33×10^6^ | 5.11×10^4^ | 8.60×10^4^ | 9.30×10^4^ |
| rectum | / | / | / | / | / | / | 3.52×10^7^ | 1.64×10^8^ | 1.36×10^5^ | 3.73×10^6^ | 1.71×10^1^ | 1.79×10^4^ |

/：undetected.
